# Identifying and targeting cancer stem cells in leiomyosarcoma: prognostic impact and role to overcome secondary resistance to PI3K/mTOR inhibition

**DOI:** 10.1186/s13045-018-0694-1

**Published:** 2019-01-25

**Authors:** Benjamin Fourneaux, Aurélien Bourdon, Bérengère Dadone, Carlo Lucchesi, Scott R. Daigle, Elodie Richard, Audrey Laroche-Clary, François Le Loarer, Antoine Italiano

**Affiliations:** 1 Université de Bordeaux, Bordeaux, France; 2 Institut National de la Santé et de la Recherche Medicale (INSERM) U1218, Institut Bergonié, 229 Cours de l’Argonne, 33000 Bordeaux, France; 3 Department of Pathology, Nice University Hospital, Nice, France; 4 Epizyme, Inc., Cambridge, MA USA; 5 Department of Medical Oncology, Institut Bergonié, Bordeaux, France

**Keywords:** Leiomyosarcoma, BEZ235, Cancer stem cell, ALDH1, EZH2

## Abstract

**Background:**

Leiomyosarcoma (LMS) is one of the most frequent soft tissue sarcoma subtypes and is characterized by a consistent deregulation of the PI3K/mTOR pathway. Cancer stem cells (CSCs) have been poorly studied in soft tissue sarcomas. In this study, we aimed to evaluate the association between CSCs, the outcome of LMS patients, and the resistance to PI3K/mTOR pathway inhibition.

**Methods:**

We investigated the relationships between aldehyde dehydrogenase 1 (ALDH1) expression, a cancer stem cell marker, and the outcome of LMS patients in two independent cohorts. We assessed the impact of CSCs in resistance to PI3K/mTOR pathway inhibition using LMS cell lines, a xenograft mouse model, and human tumor samples.

**Results:**

We found that enhanced ALDH1 activity is a hallmark of LMS stem cells and is an independent prognostic factor. We also identified that secondary resistance to PI3K/mTOR pathway inhibition was associated with the expansion of LMS CSCs. Interestingly, we found that EZH2 inhibition, a catalytic component of polycomb repressive complex which plays a critical role in stem cell maintenance, restored sensitivity to PI3K/mTOR pathway inhibition. Importantly, we confirmed the clinical relevance of our findings by analyzing tumor samples from patients who showed secondary resistance after treatment with a PI3Kα inhibitor.

**Conclusions:**

Altogether, our findings suggest that CSCs have a strong impact on the outcome of patients with LMS and that combining PI3K/mTOR and EZH2 inhibitors may represent a promising strategy in this setting.

**Electronic supplementary material:**

The online version of this article (10.1186/s13045-018-0694-1) contains supplementary material, which is available to authorized users.

## Background

Leiomyosarcomas (LMSs) are one of the most frequent histological subtypes of soft tissue sarcomas (15–20%). The prognosis for LMSs is poor, with up to 40% of patients experiencing metastatic relapse despite optimal locoregional treatment.

LMSs belong to the class of sarcomas with complex genomic alterations characterized by non-recurrent structural and copy number alterations [1]. In 40–50% of LMS cases, cytogenetic studies have shown a loss of chromosome 10q encompassing *PTEN* (phosphatase and tensin homolog), a tumor suppressor gene and a negative regulator of phosphoinositide 3-kinase (PI3K) [2, 3]. Conditional knockout of *PTEN* from the smooth muscles of mice predisposes them to the development of LMSs in various organs [4]. Strikingly, a recent study conducted by The Cancer Genome Atlas (TCGA) showed a correlation of PTEN alteration with a very high signaling of the PI3K/mTOR pathway in LMS characterized by amplifications or overexpressions of different genes regulating the pathway [5]. Our group and others have reported that dual PI3K and mTOR inhibition is associated with strong anti-tumor activity in LMS, which was significantly greater than that of either mTOR inhibition or PI3K inhibition alone [6, 7].

While several dual PI3K/mTOR inhibitors are under development, this class of drugs suffers from the same major limitation associated with other targeted therapies and traditional chemotherapy drugs in a metastatic disease setting; that is, the duration of any observed clinical benefit is limited, owing to the relatively rapid acquisition of drug resistance. Therefore, identifying specific molecular mechanisms of resistance is crucial to define new strategies to overcome or prevent the development of resistance to PI3K/mTOR inhibitors in the clinical setting.

Cancer stem cells (CSCs) have been widely investigated in a range of hematopoietic and epithelial tumors. There are several lines of evidence indicating that CSCs represent a crucial mechanism of resistance to anti-cancer drugs [8]. However, CSCs have been poorly studied in sarcomas. We report here the first study identifying CSCs in LMS, assessing their prognostic impact on the outcome and their role in resistance to therapy, and describe for the first time how an epigenetic intervention may reverse their phenotype and improve response to therapy.

## Methods

### Cell culture

Leiomyosarcoma cell lines were obtained and established as previously described [6]. To generate BEZ235-resistant cell lines, parental cells were cultured with increasing concentrations of BEZ235 starting with a concentration of 0.1 nM. Fresh drug was added every 72 h. Resistant cells were maintained as polyclonal populations under constant 50 nM BEZ235 selection. Microarray-based comparative genomic hybridization (aCGH) analysis of both the parental and resistant cells confirmed that the cells were derived from the same origin. For details including drugs used, growth and apoptosis assays, and western blotting, see the Methods section in Additional file 1.

### Clinical samples

Tissue microarray (TMA) was used to study the immunohistochemistry (IHC) expression of ALDH1 and p-S6 in two distinct cohorts of LMS (cohort A *n* = 145; cohort B *n* = 89) treated at the Institut Bergonié (Bordeaux, France) (Additional file 1: Table S1). The staining intensity was scored as follows: 1 (weak), 2 (moderate), or 3 (strong). The proportion of positive cells was also evaluated. Scores for both parameters (staining intensity and proportion of positive cells) were multiplied to generate an immunohistochemistry score, e.g., if the reactivity score was 3 (strong) and the proportion of positive cells was 3 (26–50%), the final immunohistochemistry score is given by 3 × 3 = 9.

Three patients treated with an investigational PI3Kα inhibitor (BYL719) had available tumor material collected before treatment onset and at the occurrence of disease progression.

### Sequencing

The RNAseq of LMS cell lines and patient samples was performed by GATC Biotech (Konstanz, Germany) using standard Illumina (Illumina Inc., San Diego, CA, USA) protocols using the HISeq2500 sequencing engine. For detailed analysis methods, see the Additional file 1.

### Heatmap

Unsupervised clustering was realized using hclust function [9]. Pearson’s correlation was used as similarity criteria, then the average linkage agglomerative method was used to define observed clusters. To construct a heatmap based on normalized gene expression means, the “heatmap” R function develop by Andy Liaw was used.

### Aldefluor assay

The Aldefluor Kit (Stem Cell Technologies, Durham, NC, USA) was used to detect stem cells with high ALDH1 enzyme activity according to the manufacturer’s instructions. Based on this activity, a sphere-forming assay was assessed. For details, see the Methods section in Additional file 1.

### Animal studies

For in vivo experiments, LMS cells (5 × 10^6^ cells/100 μL) were injected subcutaneously into the right flank of Ragγ2C−/− mice (*n* = 8). Three weeks after drug administration, mice were euthanized and tumors were excised. For details, see the Methods section in Additional file 1.

### Study approval

The study was approved by the Institutional Review Board of the Institut Bergonié (Bordeaux, France), and the methods were carried out in accordance with the approved guidelines and with written informed consent from all patients. All animal experiments under project license APAFIS 8414 were performed with the approval of the Institutional Animal Use and Care Committee**.**

## Results

### Cancer stem cell marker aldehyde dehydrogenase has independent prognostic value in LMS

**Fig. 1:**
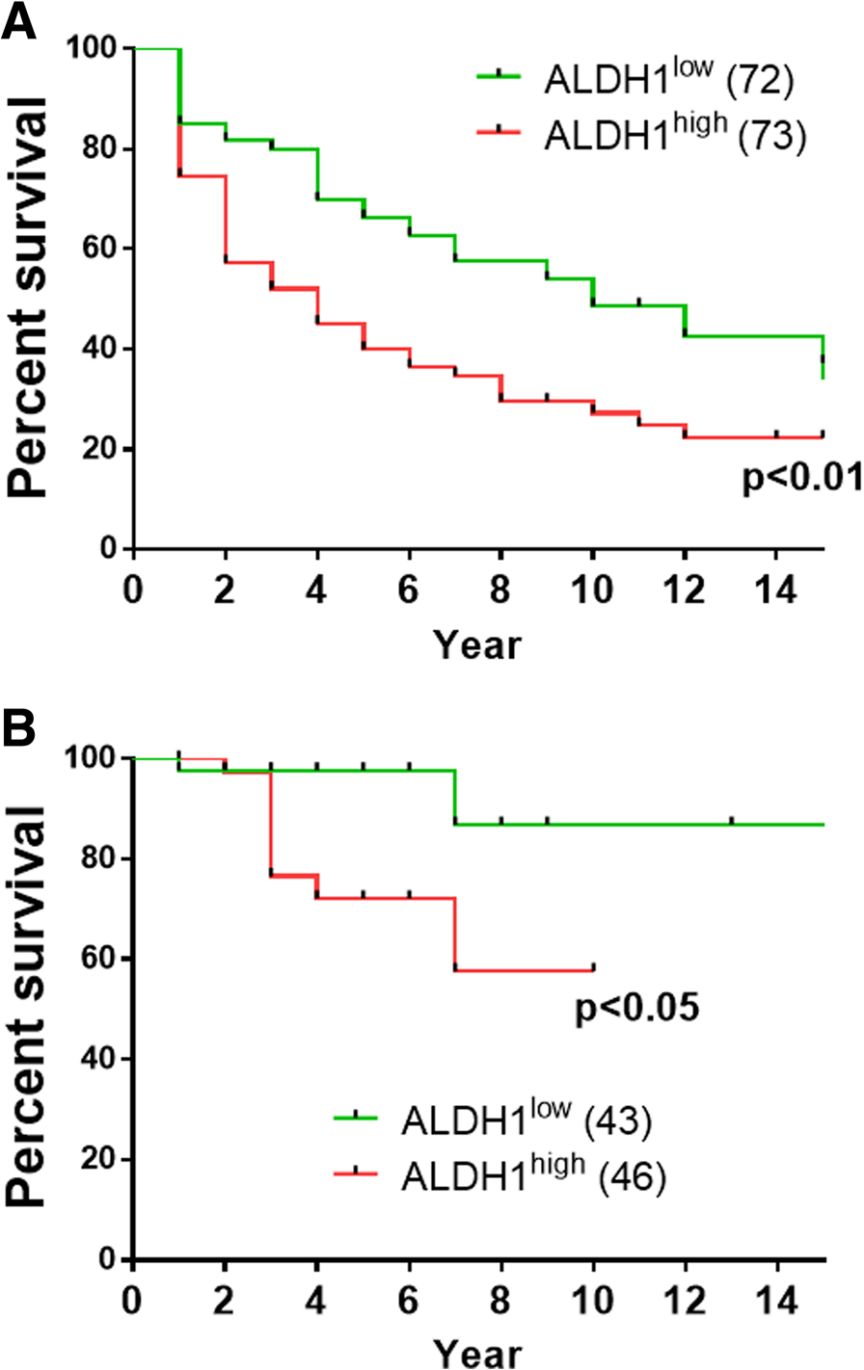
Study of expression of ALDH1 in patients with sarcoma. **a** Kaplan-Meier curves of overall survival according to expression levels of ALDH1 from 145 LMS. **b** Kaplan-Meier curves of overall survival according to expression levels of ALDH1 from ICGC cohort (89 LMS). Red line, expression higher than the median expression level; green line: expression lower than the median. The result of the logrank test is indicated in each graph

Elevated aldehyde dehydrogenase 1 (ALDH1) is considered a universal marker of CSCs [10]. Studies investigating bone sarcoma cell lines indicated that ALDH1^high^ cells had increased capacity to form spheres and colonies and had significantly greater frequency of tumor-initiating cells [11, 12]. We have therefore analyzed by using IHC the expression of the ALDH isoform 1 expression in two independent series of LMS (cohort 1 *n* = 145, cohort 2 *n* = 89) as shown in Fig. 1a, b. On multivariate analysis, high ALDH1 expression score was significantly associated with poor metastases-free survival (hazard ratio = 1.5 [95% CI 1.3–1.8], *p* = 0.04), together with grade 3 (hazard ratio = 1.5 [95% CI 1.3–1.8], *p* = 0.004), and a tumor size of > 10 cm (hazard ratio = 1.5 [95% CI 1.3–1.8], *p* = 0.001). We did not find any correlation between the level of ALDH1 expression and the level of p-S6 expression (data not shown).

### Generating dual PI3K/mTOR inhibitor resistant LMS cells in vitro and in vivo

**Fig. 2:**
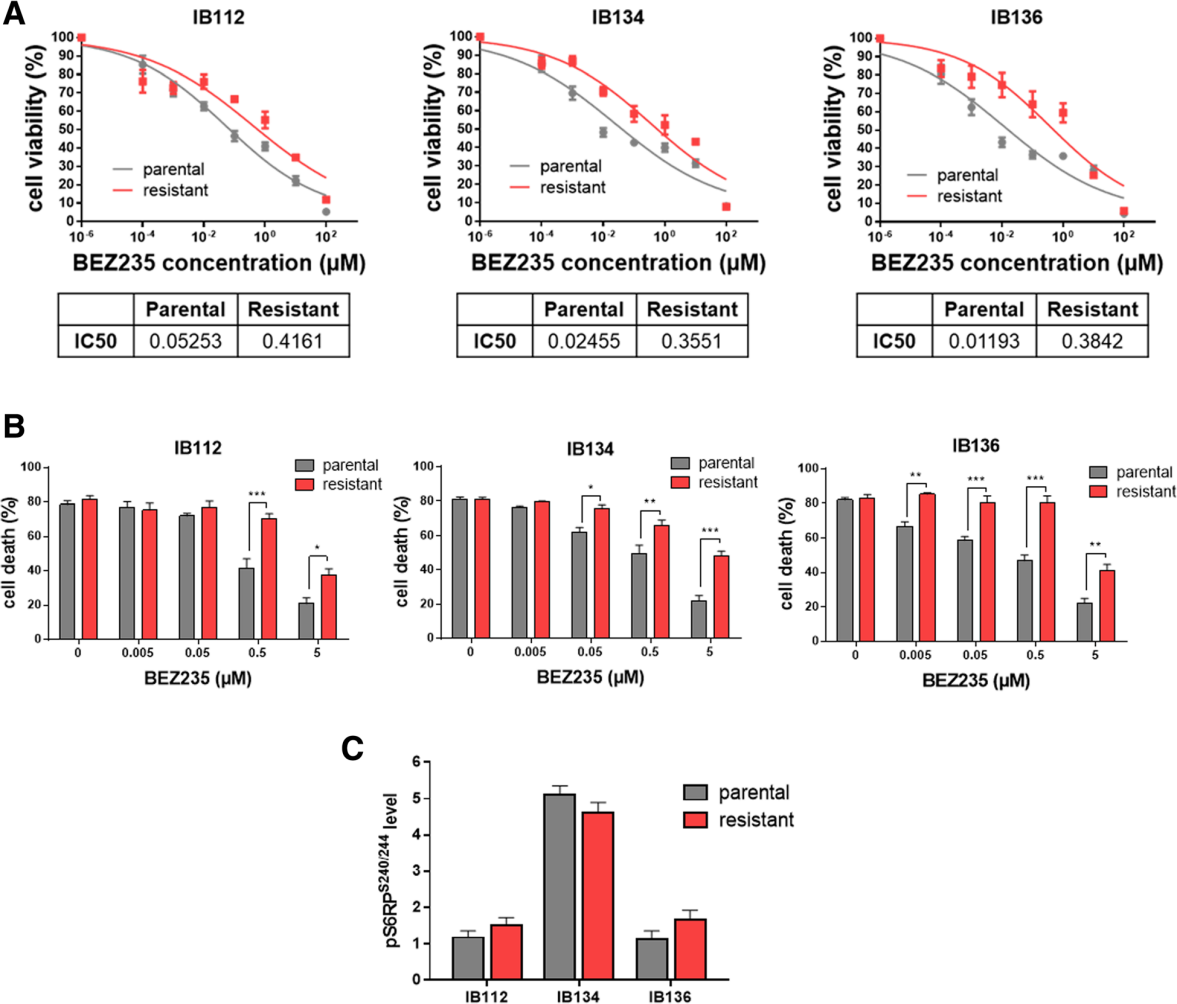
Anti-tumor effect of dual PI3K/mTOR inhibition in parental and resistant LMS cell lines. **a** Growth curves indicating growth inhibition of the three parental (IB112, IB134, and IB136) and resistant (resIB112, resIB134, and resIB136) LMS cell lines after BEZ235 treatment for 72 h. IC_50_ is indicated in μM. **b** Percentage of apoptotic cells after BEZ235 treatment for 72 h. Data are presented as the mean ± SEM of three independent experiments. *resistant *p* < 0.05 vs. parental; **resistant *p* < 0.01 vs. parental; ***resistant *p* < 0.001 vs. parental (two-way ANOVA). **c** Representative signal intensities, obtained by western blotting, for p-S6RP^S240/244^ were normalized to those for total S6RP in each cell line. Data are presented as the mean ± SEM of three independent experiments

In order to confirm that enhanced ALDH1 activity is a hallmark of LMS stem cells and the potential role of these cells in resistance to therapy, we decided to generate resistant LMS cells from three LMS cell lines we have previously described to be sensitive to dual PI3K/mTOR inhibition [6]. We exposed these cell lines were to BEZ235, a dual PI3K/mTOR inhibitor, using doses that were increased in a stepwise manner, and surviving cells were selected until normal cell growth resumed. Thus, we independently established three BEZ235-resistant LMS cell lines (resIB112, resIB134, and resIB136), which exhibited BEZ235 IC_50_ values that were > 8-fold higher than the parental cell line (Fig. 2a). Cells required approximatively 48 weeks in culture to achieve resistance. As indicated in Fig. 2b, secondary resistant cells were significantly more resistant than parental cells to BEZ235-induced apoptosis. No cross-resistance with other anti-cancer agents approved for the management of sarcoma patients (doxorubicin, gemcitabine) was observed (Additional file 1: Figure S1A and S1B). We further explored the PI3K/mTOR pathway by western blotting using phosphorylation of S6RP as a marker of pathway activation. No change in pS6RP^S240/244^ accumulation was observed between the three parental and resistant LMS cell lines (Fig. 2c).

**Fig. 3:**
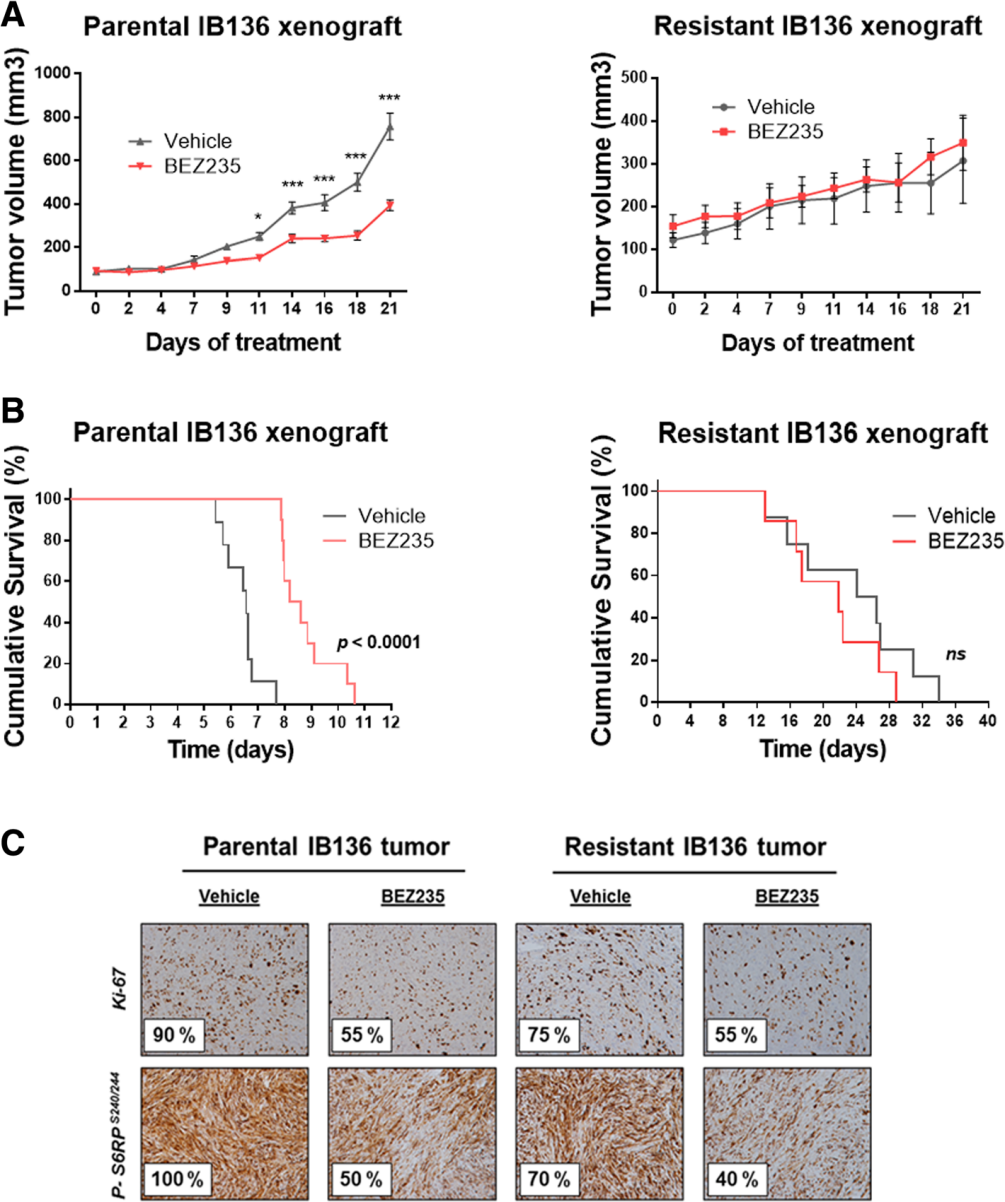
Anti-tumor effect of dual PI3K/mTOR inhibition in IB136 and resIB136-derived xenografts in Ragγ2C−/− mice. **a** Tumor volume progression curves over 3 weeks of BEZ235 treatment. Mice were randomly assigned to receive 40 mg/kg of BEZ235 or vehicle. The data points represent an average from 8 mice (bars, SEM). *p < 0.05; ***p < 0.001, two-way ANOVA. **b** Kaplan-Meier curves for tumor doubling times. **c** Immunohistochemical staining images of tumor samples treated with anti-Ki-67 and anti-pS6RP^ser240/244^ antibodies (objective magnification, × 10). Percentages correspond to positively stained cells estimated by a pathologist

These in vitro findings prompted us to examine whether these cells were drug resistant in an in vivo setting. Thus, IB136 and resIB136 xenografts were established and grew to a size of 100 mm^3^, after which mice were treated orally with either vehicle or BEZ235 5 days a week for 3 weeks. Our results showed that 40 mg/kg of BEZ235 treatment potently inhibited tumor growth of IB136-derived xenografts (55% tumor inhibition, *p* < 0.05), while tumor growth of resIB136-derived xenografts was strongly similar to the vehicle-treated group (Fig. 3a). Also, BEZ235 treatment highly significantly slowed the rate of parental IB136 tumor growth compared to the control (8.4 vs. 6.5 days for median survival; *p* < 0.0001) and had no effect on resIB136-derived xenograft growth as shown in Fig. 3b. No apparent toxicity events were observed in the drug-treated animals. There were no significant changes in animal weight (data not shown). The number of tumor cells positive for Ki-67, a cell proliferation marker, and p-S6RP^s240/244^, a protein indicating activity of the PI3K/mTOR pathway, was substantially lower in IB136 and resIB136 xenografts treated with BEZ235 compared with control tumors (Fig. 3c). Altogether, these results showed BEZ235 could inhibit PI3K/mTOR signaling pathway of both tumors but had no effect on resIB136 tumor growth suggesting, in vivo, the presence of an acquired-resistance to treatment.

### Cancer stem cell (CSC) markers were upregulated in LMS tumors with acquired resistance to dual PI3K/mTOR inhibition

**Fig. 4:**
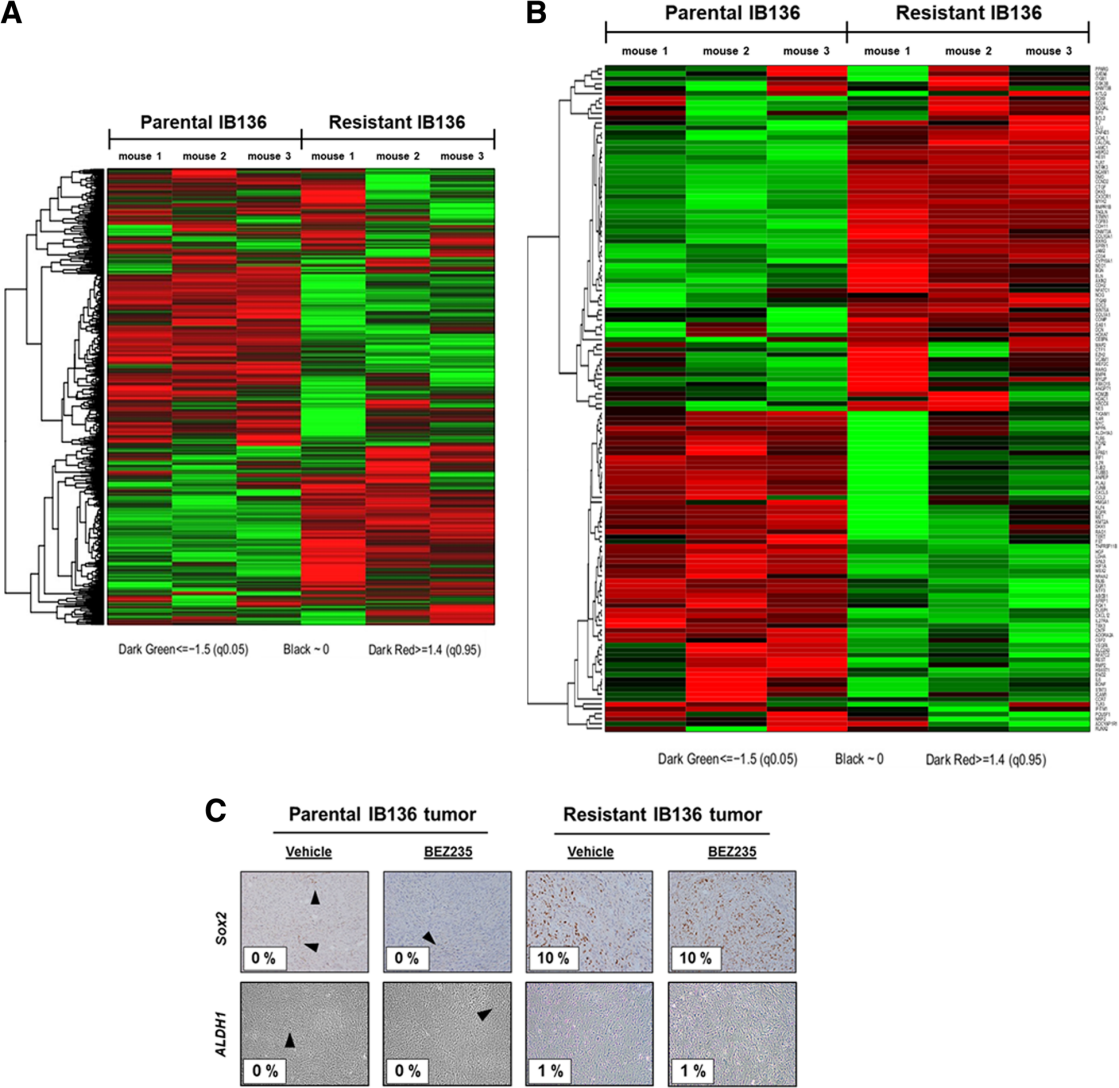
Heatmap of differentially expressed genes between the IB136 and resIB136-derived xenografts. **a** Heatmap based on mean, normalized expression of general differentially expressed genes. **b** Heatmap based on mean, normalized expression of stem cell signaling components. The red and green colors denote high and low intensities, respectively. **c** Immunohistochemical staining images of tumor samples treated with anti-SOX2 and anti-ALDH1 antibodies (objective magnification, × 10). Percentages correspond to positively stained cells estimated by a pathologist. Endothelial cells (positive control) are indicated by black arrows

To identify the mechanisms of secondary resistance to dual PI3K/mTOR inhibition, we performed whole-transcriptome sequencing (RNAseq) of three independent parental and resistant tumors. We identified 985 genes that were differentially expressed between IB136-derived parental and resistant tumor xenografts, among which 583 genes were upregulated, and 402 genes were downregulated (with two times or more fold-change and an adjusted *p* value < 0.01). Features of differentially expressed genes in resIB136 tumors were summarized (upregulated genes in Additional file 1: Table S2 and downregulated genes in Additional file 1: Table S3). Afterwards, the limma and GSEA were performed to evaluate the different gene expression and pathways between these two groups. The heatmap showed that there was a distinct gene expression pattern between the IB136-derived parental and resistant tumor xenografts (Fig. 4a). The results showed that these differentially expressed genes were highly enriched in proliferative, growth, and embryonic development networks (Additional file 1: Table S4). Transcription levels of most molecules in stem cell pathway are either continuously upregulated, downregulated, or unaffected (Fig. 4b). When analyzing the differentially expressed genes of this pathway, we found in the resistant group, NCAM1, as the most strongly upregulated gene with a fold-change of 375.15 (Additional file 1: Table S5). Moreover, upregulation of *ALDH1A2* (fold-change of 10.40), *KLF5* (fold-change of 6.73), and *SOX2* (fold-change of 2.5) suggested that secondary resistance to dual PI3K/mTOR inhibition could be associated with the emergence of a CSC-like subpopulation. Indeed, these markers have been reported as being involved in the self-renewal and self-protection of cancer stem cells [13, 14]. To confirm our hypothesis at the protein level, we examined the immunohistochemical expression of the stem cell marker SOX2 and ALDH1 in the parental and secondary resistant xenografts and found significantly higher expression in the resistant tumors (Fig. 4c).

### LMS cells with ALDH1 activity have tumorigenic and resistant CSC properties

**Fig. 5:**
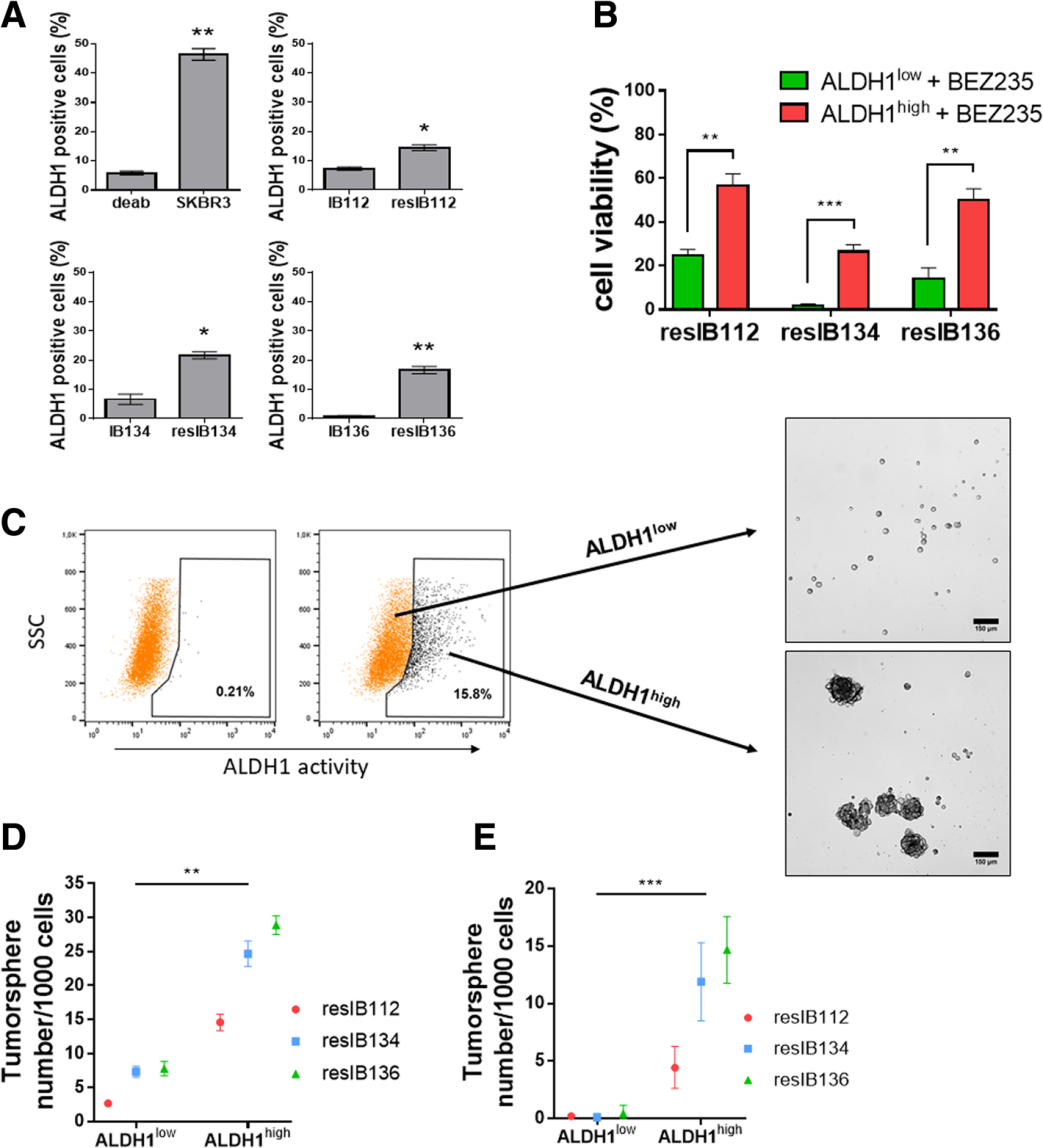
Measurement of ALDH1 activity and its role in tumorigenicity and resistance. **a** Percentage of parental and resistant cells with ALDH1 activity as determined by flow cytometry. The SKBR3 cell line represents a positive control in the Aldefluor assay. Data are presented as the mean ± SEM of three independent experiments. *resistant *p* < 0.05 vs. parental; **resistant *p* < 0.01 vs. parental (two-tailed Student’s *t* tests). **b** Percentage of viable ALDH1^high^ and ALDH1^low^ FACS-sorted BEZ235-resistant cells after 72 h of 0.3 μM of BEZ235 treatment. The results represent the mean ± SEM. **p < 0.01; ***p < 0.001, one-way ANOVA. **c** Resistant LMS cells were sorted by FACS based on ALDH1 activity and the positive and negative subpopulations of sorted cells were submitted to in vitro and in vivo tumorsphere assays. Representative images of cell sorting by FACS and phase contrast microscopy of tumorspheres formed by resIB136 cells after 15 days of culture in nonadherent conditions in vitro. Scale bars, 150 μM. **d** Number of tumorspheres formed after 15 days in ALDH1^high^ and ALDH1^low^ subpopulation. **e** Number of tumorspheres formed after 15 days by cells dissociated from tumorspheres. The results represent the mean ± SEM. **p < 0.01; ***p < 0.001, one-way ANOVA

Then, to examine whether secondary resistant LMS cell lines contain a CSC-like subpopulation compared to parental cell lines, we performed the Aldefluor assay in parental and resistant cell lines. Several lines of evidence showed that enhanced ALDH1 activity, measurable by the Aldefluor assay, is a hallmark of cancer stem cells [15]. Flow cytometry analyses revealed that ALDH1 activity was significantly higher in resistant cell lines compared to parental lines (Fig. 5a). These results indicated that the three BEZ235-resistant cell lines contain a subset of cells with high ALDH1 enzymatic activity.

CSCs possess an increased capacity for tumorigenicity and resistance to anti-cancer drugs. We hypothesized that ALDH1^high^ cells are CSCs. To further address the characteristics of these high-ALDH1 LMS cells and their role in resistance to dual PI3K/mTOR inhibition, we isolated ALDH1^low^ and ALDH1^high^ subpopulations from the resistant LMS cell lines by using fluorescence activated cell sorting (FACS). We then compared their respective viability after 72 h of treatment with 5 μM BEZ235 (Fig. 5b). The ALDH1^high^ subpopulation was significantly more refractory to BEZ235 than was the ALDH1^low^ subpopulation in all the three cell lines. To evaluate the stem cell properties of the LMS cells according to the level of ALDH1 expression, we submitted ALDH1^low^ and ALDH1^high^ subpopulations to a tumorsphere assay (Fig. 5c). In the three resistant leiomyosarcoma cell lines, ALDH1^high^ cells formed significantly more tumorspheres after 15 days of in vitro culture than their respective negative counterparts (Fig. 5d). A self-renewal assay with cells taken from the tumorspheres confirmed that the ALDH1^high^ subpopulation was comprised of tumorigenic CSCs (Fig. 5e). To confirm these results in vivo, xenografts were performed in mice with resIB112-, resIB134-, and resIB136-FACS-sorted cells based on ALDH1 activity (Additional file 1: Table S6). In all cases, tumors developed at a significant higher frequency in ALDH1^high^ cells than in their respective ALDH1^low^ cells (cancer-initiating cell frequencies range from 1/144 to 1/3104 for ALDH1^high^ cells vs. 1/1514 to 1/21,465 for ALDH1^low^ cells, *p <* 0.05). Altogether, these results indicate that ALDH1 is useful for the detection and isolation of CSCs with tumorigenic and resistant properties in leiomyosarcoma.

### Pretreatment of resistant LMS cells with an EHZ2 inhibitor significantly re-sensitizes cells to BEZ235 in vitro and in vivo

**Fig. 6:**
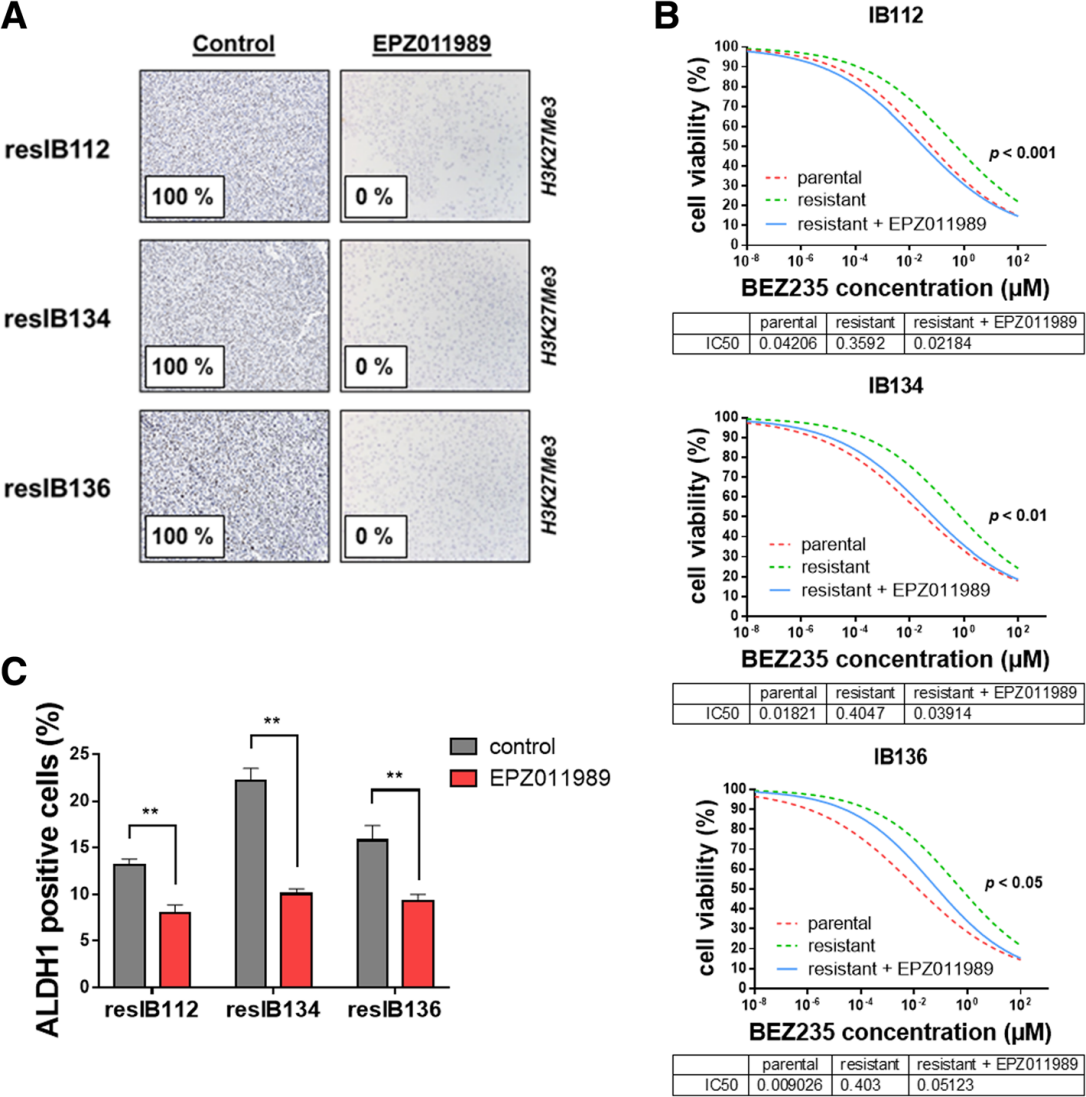
Effect of EPZ011989 pretreatment on dual PI3K/mTOR sensitivity and on the CSC-like subpopulation in resistant leiomyosarcoma cell lines. **a** Immunohistochemical staining images of BEZ235-resistant LMS cell line pellets treated with anti-H3K27Me3 antibody (objective magnification, × 10). Percentage corresponds to positively stained cells estimated by a pathologist. **b** Growth curves indicating growth inhibition of the three resistant (resIB112, resIB134, and resIB136) LMS cell lines after BEZ235 treatment for 72 h. Resistant + EPZ011989 are the cells which were pretreated for 1 week with 10 μM of EPZ011989. IC_50_ is indicated in μM. *p* value was calculated by one-way ANOVA. **c** Percentage of cells with ALDH1 activity, as determined by flow cytometry on resistant cell lines exposed to 10 μM of EPZ011989 for 1 week. Data are presented as the mean ± SEM of three independent experiments. **p < 0.01, two-tailed Student’s *t* tests

Since the polycomb repressive complex 2 (PRC2), including EZH2, has been shown to play a crucial role in stem cell maintenance [16, 17], we decided to investigate whether secondary resistance to PI3K/mTOR pathway inhibition observed in vitro and in vivo was correlated with PRC2 activity. When PRC2 is activated, it catalyzed the trimethylation of lysine 27 of histone H3 (H3K27Me3). Indeed, the use of a potent orally available EZH2 inhibitor, EPZ011989, showed a marked decrease in the number of H3K27Me3-positive cells compared with the control (Fig. 6a). Therefore, we wondered whether blocking PRC2 activity with an EZH2 inhibitor could restore sensitivity to dual PI3K/mTOR inhibition. To this end, we pretreated BEZ235-resistant cells with EPZ011989 for 1 week followed by treatment with BEZ235 for 72 h. Pretreatment of resistant cells with EPZ011989 restored significatively the sensitivity to BEZ235 (*p* < 0.05), generating IC_50_ values that were similar to the parental cell lines (Fig. 6b). Moreover, flow cytometric results revealed that ALDH1 activity was decreased in resistant cells pretreated with EPZ011989 compared to untreated resistant cells suggesting that EZH2 inhibition activity can reduce the presence of the CSC-like subpopulation in the tumor (Fig. 6c). This effect was confirmed on the formation of tumorsphere by both resistant cell lines, in which EPZ011989 treatment, alone or in combination with BEZ235 treatment, reduced significantly the number of tumorigenic CSCs (Additional file 1: Figure S2A and S2B) while BEZ235 treatment at IC_50_ only slightly affected the capacity of ALDH1^high^ cells to form spheres.

**Fig. 7:**
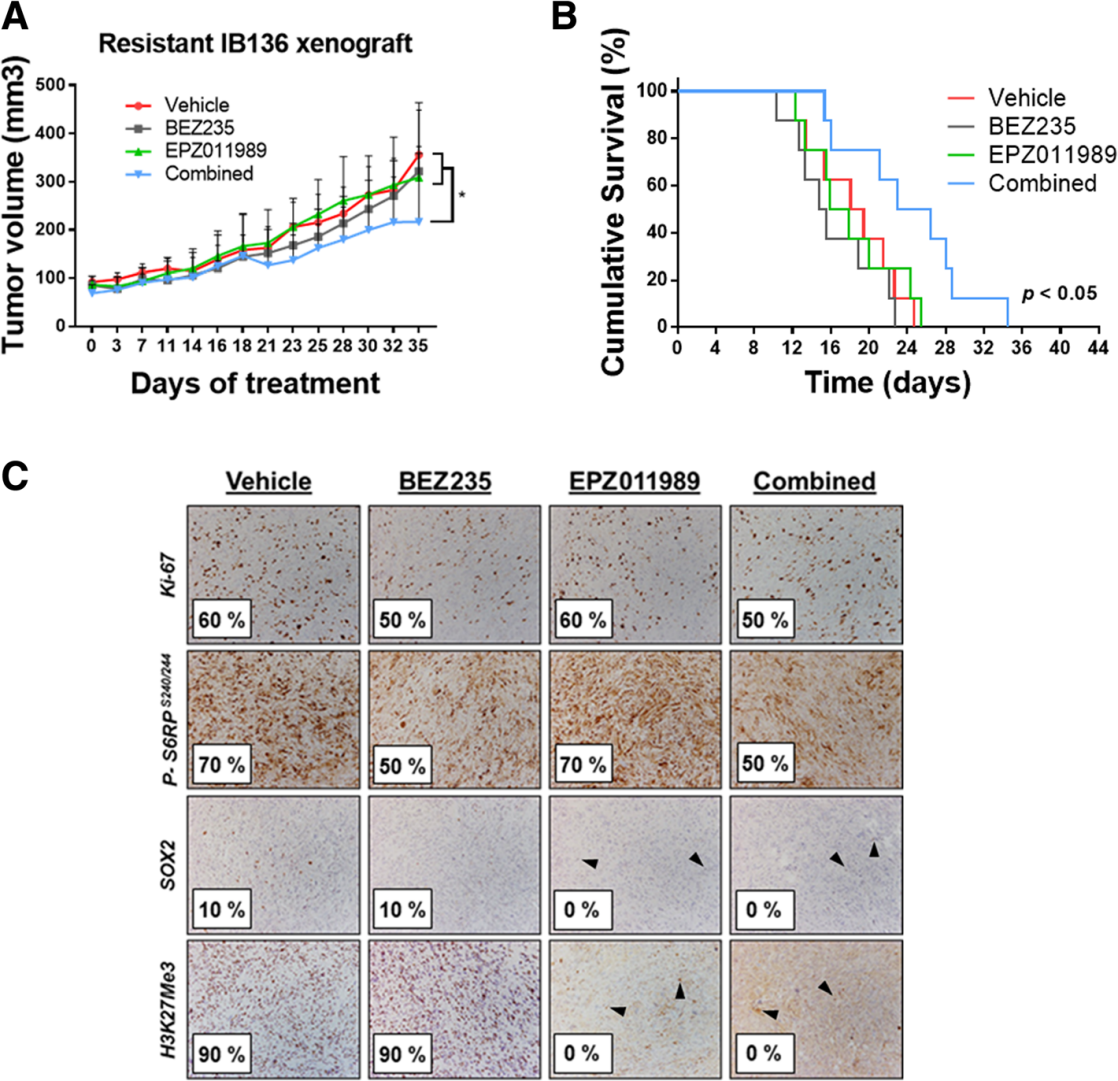
Effect of BEZ235 and EPZ011989 on resIB136-derived xenografts in Ragγ2C−/− mice and study of expression of H3K27Me3 in patients with sarcoma. **a** Tumor volume progression curves during 5 weeks of treatment. Mice were randomly pretreated with vehicle or 125 mg/kg of EPZ011989 BID for 2 weeks. Then, in the EPZ011989-pretreated group, mice were randomly treated for 3 weeks with 40 mg/kg of BEZ235 or 125 mg/kg of EPZ011989 BID. The data points represent an average from eight mice (bars, SEM). *p < 0.05, two-way ANOVA. **b** Kaplan-Meier curves for tumor doubling times. **c** Immunohistochemical staining images of tumor samples treated with anti-Ki-67, anti-pS6RP^ser240/244^, anti-SOX2, and anti-H3K27Me3 antibodies (objective magnification, × 10). Percentages correspond to positively stained cells estimated by a pathologist. Endothelial cells (positive control) are indicated by black arrows

Moreover, we tested whether PRC2 blockade could re-sensitize cells to dual PI3K/mTOR inhibition in vivo. resIB136 xenografts were established and allowed to grow to a size of 100 mm^3^. Mice were randomly pretreated with either vehicle or 125 mg/kg of EPZ011989 BID for 2 weeks. Then, in the EPZ011989-pretreated group, mice were randomly treated for 3 weeks with 40 mg/kg of BEZ235 or 125 mg/kg of EPZ011989 BID. The EPZ011989 pretreatment plus BEZ235 group showed an anti-tumor effect with a significant (*p* < 0.05) reduction of tumor growth (average tumor volume at endpoint, 216.5 ± 50.3 mm^3^) compared with either drug alone (321.8 ± 51.75 mm^3^ for BEZ235 and 308.9 ± 55.02 mm^3^ for EPZ011989) or vehicle (355.9 ± 73.83 mm^3^) as shown in Fig. 7a. Additionally, the survival data of mice (from the first day of the experiment until the day when the tumor size doubled) showed that the combination treatment significantly (*p* < 0.05) slowed the rate of tumor growth (24.65 days for median survival) compared to the vehicle (18.76 days) and the individual drug (15.12 days for BEZ235 and 16.89 days for EPZ011989) treatment groups (Fig. 7b). No apparent toxicity events were observed in the drug-treated animals. There were no significant changes in animal weight (data not shown). Further immunohistochemical analyses showed that the number of tumor cells positive for p-S6RP^s240/244^ was substantially lower in tumors treated with BEZ235. None of the tumors pretreated with EPZ011989 displayed SOX2- or H3K27Me3-positive cells (Fig. 7c). Altogether, these results showed that pretreatment of EPZ011989 could affect the differentiation of CSC-like subpopulation to re-sensitize the dual PI3K/mTOR inhibitor.

### Clinical validation of the presence of a CSC-like subpopulation as a potential resistance mechanism to a PI3K/mTOR pathway inhibitor

**Fig. 8:**
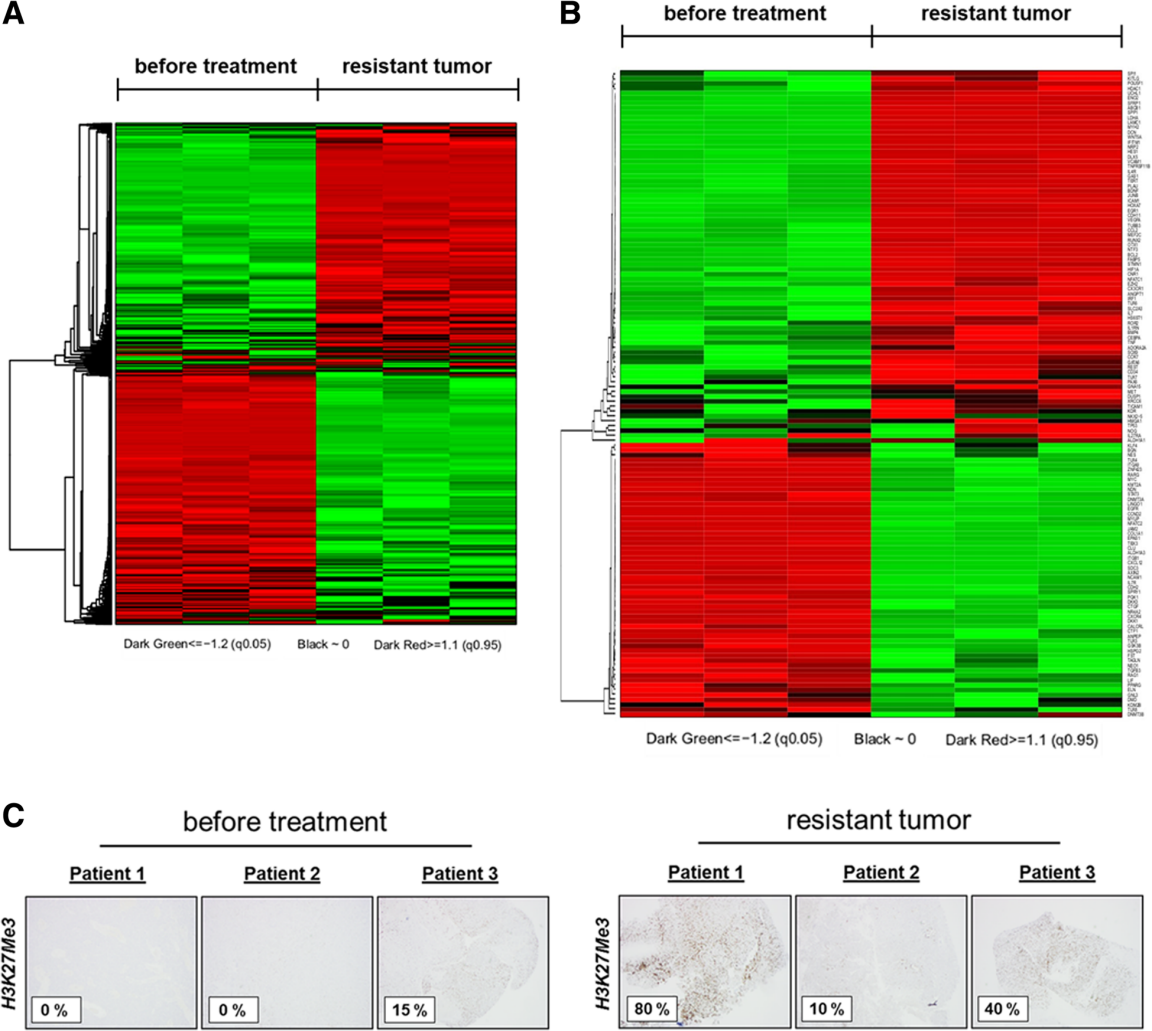
Study of expression of H3K27Me3 and heatmap of differentially expressed genes in patients with sarcoma. **a** Heatmap based on mean, normalized expression of general differentially expressed genes. **b** Heatmap based on mean, normalized expression of stem cell signaling components. The red and green colors denote high and low intensities, respectively. **c** Immunohistochemical staining images of three pairs of primary and PI3Kα inhibitor-resistant sarcoma tumor samples treated with anti-H3K27Me3 antibody (objective magnification, × 10). Percentages correspond to positively stained cells estimated by a pathologist. Endothelial cells (positive control) are indicated by black arrows

To confirm our in vitro and in vivo observations, we decided to analyze tumor samples from three patients treated with an investigational PI3Kα inhibitor and for whom tumor material was collected prior to treatment onset and at occurrence of secondary resistance. We were able to perform whole-transcriptome sequencing (RNAseq) of paired primary/secondary resistant tumor samples from one of these three patients. Then, 1110 genes were differentially expressed in the resistant tumor, among which 991 genes were upregulated and 119 genes were downregulated (with four times or greater fold-change and an adjusted *p* value < 0.01). Features of differentially expressed genes in this clinical sample were summarized (upregulated genes in Additional file 1: Table S7 and downregulated genes in Additional file 1: S8). By applying the same analysis methods as described previously, limma and GSEA were performed to show the different gene expression pattern and enriched pathways between the primary and secondary resistant tumor (Fig. 8a). Strikingly, the results once again showed a significant enrichment of stem cell pathway (*p* < 10^− 4^) in the resistant tumor sample (Additional file 1: Table S9). Transcription levels of most molecules in the stem cell pathway are either continuously upregulated, downregulated, or unaffected (Fig. 8b). Of these markers, *POU5F1* and *NANOG* were upregulated with a fold-change of 38.75 and 2.1, respectively (Additional file 1: Table S10). *POU5F1*, also known as *Oct*-*3*/*4*, and *NANOG*, are one of the most important proteins associated with the pluripotent properties of stem cells and is an essential factor in controlling the early stages of mammalian embryogenesis [18]. We decided to investigate whether secondary resistance to PI3Kα inhibition in these patients was correlated with PRC2 activity. Interestingly, by using IHC, we found a significantly higher level of H3K27Me3 in resistant tumors compared with primitive tumors (Fig. 8c).

## Discussion

We have demonstrated here that high ALDH1 activity is a very strong marker of LMS CSCs and that expression of ALDH1 is an independent prognostic factor in patients with LMS. Given the importance of the PI3K/mTOR pathway in the pathogenesis of LMS, we sought to identify mechanisms of resistance to targeted PI3K/mTOR inhibition in LMS models and the potential role of CSC. To this end, we used our recently characterized LMS cell lines [6], xenograft models, and patient tumor samples. Although each model contains inherent limitations, we have gained appreciable insight into the potential underlying mechanisms of resistance that could be anticipated in response to targeted inhibition of the PI3K/mTOR axis in this disease segment. The models that we used acquired resistance in vitro after 48 weeks of increasing drug pressure. Importantly, we were able to confirm our in vitro resistance models in vivo*,* and the resistance phenotype observed was specific to dual PI3K/mTOR inhibition due to lack of cross-resistance with other commonly used LMS drugs such as doxorubicin or gemcitabine.

In our model, secondary resistance was associated with the expansion of a subpopulation of cells with stem-like characteristics, including enhanced ALDH1 activity. Importantly, we were able to demonstrate the stem-cell-like nature of this subpopulation and its involvement in resistance by several methods: (1) genomic profiling of parental and resistant tumors using RNAseq paired with gene set-enrichment analysis which to identify the upregulation of several stem cell genes, (2) isolation of ALDH1^high^ cell subpopulations by using the fluorescent reagent (ALDEFLUOR®, Stem Cell Technologies, Durham, USA), (3) evaluation of the toxicity of dual PI3K/mTOR inhibition on ALDH1^low^ and ALDH1^high^ cells, and (4) assessment of the ability of the ALDH1^high^ subpopulation to form spherical colonies when grown under non-adherent conditions, to self-renew and to form a new tumor after xenograft in vivo.

Several studies have shown that epigenetic modifications, including those induced by polycomb group (PcG) proteins, play a crucial role in CSC maintenance. Indeed, epigenetic modification by PcG proteins is critical for maintaining stem-cell-like characteristics in adult stem cells and in embryonic stem cells [19]. As a key protein in the PcG family, EZH2 is the catalytic subunit of the multi-protein histone methyltransferase complex known as PRC2. The core catalytic complex is composed of four different proteins: EZH2, EED, SUZ12, and RBAP48. PRC2 catalyzes the mono-, di-, and trimethylation of H3K27. The trimethylated form of H3K27Me3 is associated with the repression of genes important for differentiation. SWI/SNF is another multi-protein complex involved in chromatin remodeling. It antagonizes PRC2 activity in regulating self-renewal and differentiation of cells [20]. In stem or progenitor cells, EZH2 activity is high and the expression of PRC2 target genes is therefore repressed. When EZH2 activity is downregulated, PRC2 target gene expression is increased through augmented SWI/SNF activity, and cells can differentiate and become quiescent. As an illustration of the importance of this complex in soft tissue sarcomas, we have recently shown that the first-in-class, first-in-human highly specific inhibitor tazemetostat (an EZH2 inhibitor) is associated with an excellent safety profile and promising anti-tumor activity in SMARCB1-deleted sarcomas [21]. Here, we have shown that EZH2 inhibition induced is able to eliminate LMS stem-like cells and to restore the anti-tumor effect of dual PI3K/mTOR inhibition in vitro and in vivo.

Importantly, we confirmed the clinical relevance of our findings by analyzing tumor samples from three patients who showed secondary resistance after treatment with a PI3Kα inhibitor. We found a significant increase in the level of H3K27Me3, the substrate of EZH2, in the samples obtained at resistance in comparison with pretreatment samples as well as a significant upregulation of several stem cell genes. This may represent a promising application of EZH2 inhibition to prevent tumor relapse as this may be caused by the drug-resistant and self-renewable sarcoma stem cells [22].

Altogether, our results provide insights into the role of CSCs on the prognosis of LMS and their sensitivity to PI3K/mTOR inhibition and consolidate the path toward novel treatment options combining inhibitors of oncogenic signaling pathways and epigenetic modulators.

## Conclusion

Altogether, our results provide insights into the role of CSCs on the prognosis of LMS and their sensitivity to PI3K/mTOR inhibition and consolidate the path toward novel treatment options combining inhibitors of oncogenic signaling pathways and epigenetic modulators.

## Additional file

Additional file 1: Supplementary Methods, **Tables (S1-S10)** and **Figures (S1-S2)**. (DOCX 1520 kb)

## Abbreviations

ALDH: Aldehyde dehydrogenase
CGH: Comparative genomic hybridization
CSC: Cancer stem cell
EZH2: Enhancer of zeste homolog 2
FACS: Fluorescence activated cell sorting
IHC: Immunohistochemistry
KLF5: Kruppel-like factor
LMS: Leiomyosarcoma
mTOR: Mammalian target of rapamycin
NCAM1: Neural cell adhesion molecule 1
PcG: Polycomb group
PI3K: Phosphoinositide 3-kinase
PRC2: Polycomb repressive complex 2
PTEN: Phosphatase and tensin homolog
RNAseq: RNA sequencing
S6RP: S6 ribosomal protein
SOX2: Sex-determining region Y-box 2
TCGA: The Cancer Genome Atlas
TMA: Tissue microarray
