# Expression of Concern to: Identifying and targeting cancer stem cells in leiomyosarcoma: prognostic impact and role to overcome secondary resistance to PI3K/mTOR inhibition

**DOI:** 10.1186/s13045-019-0825-3

**Published:** 2019-11-22

**Authors:** Benjamin Fourneaux, Aurélien Bourdon, Bérengère Dadone, Carlo Lucchesi, Scott R. Daigle, Elodie Richard, Audrey Laroche-Clary, François Le Loarer, Antoine Italiano

**Affiliations:** 1 Université de Bordeaux, Bordeaux, France; 2 Institut National de la Santé et de la Recherche Medicale (INSERM) U1218, Institut Bergonié, 229 Cours de l’Argonne, 33000 Bordeaux, France; 3 Department of Pathology, Nice University Hospital, Nice, France; 4 Epizyme, Inc., Cambridge, MA USA; 5 Department of Medical Oncology, Institut Bergonié, Bordeaux, France

**Correction to: J Hematol Oncol. 2019 12:11**

**https://doi.org/10.1186/s13045-018-0694-1**

The Editor-in-Chief would like to alert readers that the ownership of some of the data presented in this article [1] is currently under dispute. Specifically, the data in Fig. 1b is under dispute by the PI of the ICGC leiomyosarcoma program and L’Institut Bergonie. The PI of the ICGC leiomyosarcoma program has stated the authors did not have permission to use the data presented in Fig. 1b. The Board of the French Sarcoma Group, which responded to concerns from the PI of the ICGC leiomyosarcoma program, has also requested that either Fig. 1b be removed or the paper should be retracted. However, L’Institut Bergonie has stated the authors had the right to use the data and that the data were collected at the institution. Pending further institutional investigation of these concerns, further editorial action may be taken.

Aurélien Bourdon, Carlo Lucchesi, Elodie Richard, Audrey Laroche-Clary, François Le Loarer, and Antoine Italiano agree to this Expression of Concern. Benjamin Fourneaux, Bérengère Dadone, and Scott R. Daigle have not responded to any correspondence from the editor or publisher about this Expression of Concern.
