# Supplementary material for: Identifying and targeting cancer stem cells in leiomyosarcoma: prognostic impact and role to overcome secondary resistance to PI3K/mTOR inhibition
Source: J Hematol Oncol. 2019 Jan 25;12:11. doi: 10.1186/s13045-018-0694-1 (PMC6347793; doi:10.1186/s13045-018-0694-1)
Supplement: Supplementary file 1 — Supplementary Methods, Tables (S1-S10) and Figures (S1-S2). (DOCX 1520 kb) [file 13045_2018_694_MOESM1_ESM.docx]

**Additional file 1**

**Table of Contents**

[**Supplementary Methods** 2](#_Toc523247786)

[**Supplementary Figures** 8](#_Toc523247787)

[**Supplementary tables** 10](#_Toc523247788)

[**References** 20](#_Toc523247789)

# **Supplementary Methods**

**Cell culture**

The LMS cell lines IB112 and IB136 and the uterine LMS cell line IB134 were cultured in RPMI 1640 medium, GlutaMAX^TM^ Supplement (Sigma-Aldrich, St. Quentin Fallavier, France) supplemented with 10% (v/v) fetal bovine serum (FBS) and 0.2% Normocin (InvivoGen, Toulouse, France) at 37°C in an environment containing 5% CO_2_. SKBR3 cell line was purchased from ATCC and was cultured in complete Dulbecco’s Modified Eagle Medium (Sigma-Aldrich, St. Quentin Fallavier, France). Cells were routinely passaged every 2 to 3 days. The cell lines were tested negative for mycoplasma contamination.

**Reagents**

BEZ235 (dual PI3K/mTOR inhibitor) was purchased from Selleck Chemicals (Houston, TX, USA) and was prepared as a 2.1 mmol/L stock solution in DMSO and stored at -80°C. EPZ011989, a selective EZH2 inhibitor, was synthesized and provided by Epizyme Inc. (Cambridge, MA, USA). The chemotherapy drugs doxorubicin and gemcitabine were purchased from Sigma Aldrich (St. Quentin Fallavier, France). Cultured cells were treated with medium changes and fresh drug additions as indicated in the figure legends.

**Cell viability assay**

Cells were seeded in triplicate at 5000 cells/well into 96-well plates, cultured with fresh growth medium for at least 24 hours and treated with a range of increasing concentrations of BEZ235 for 72 hours. For BEZ235 and EPZ011989 combination studies, cells were pretreated for one week with a single dose of EZH2 inhibitor before seeding. Cell viability was assessed by MTT (2-deoxyglucose (2-DG) and 3-4,5-dimethylthiazol-2-yl)-2,5-diphenyltetrazolium bromide) assay (Sigma-Aldrich, St. Quentin Fallavier, France). The absorbance at 570 nm was monitored using a Flexstation 3 Plate reader (Sunnyvale, CA, USA), using 630 nm as a reference. The half maximal inhibitory concentration (IC_50_) was calculated with GraphPad Prism software version 5.00 for Windows (GraphPad Software, La Jolla, CA, USA). Each experiment was repeated at least 3 times.

**Determination of apoptosis**

LMS cells were seeded in 6-well plates and treated for 72 hours with several drug concentrations. After treatment, cells were washed once with phosphate-buffered saline (PBS) and labeled with annexin-V-FITC and propidium iodide (PI) according to the manufacturer’s protocol (BD Biosciences, San Jose, CA, USA). Then, apoptosis was determined by fluorescence-activated cell-sorting (FACS Calibur flow cytometer, BD Biosciences, San Jose, CA, USA) analysis of annexin-V-FITC and PI labelling. The percentage of cells in early apoptosis (annexin-V-positive, PI-negative) and in late apoptosis or necrosis (annexin-V- and PI-positive) was calculated using FlowJo version 7.6.3 for Windows (Tree Star Inc, Ashland, OR, USA). The percentages of overall death (sum of early and late apoptosis) are represented as the mean ± SEM values based on 3 independent experiments.

**Western blotting**

Whole cells were harvested using 60 µL radio-immunoprecipitation assay (RIPA) lysis buffer [1]. The lysate was centrifuged (13 000 rpm, 15 min, 4°C), and the supernatant was stored at -80°C until further use. Total proteins (30 µg) were electrophoresed on an 8, 12 or 15% sodium dodecyl sulfate (SDS) polyacrylamide gel and transferred onto polyvinylidene difluoride (PVDF) membranes. Blots were probed overnight at 4°C in 5% BSA (bovine serum albumin) in PBST (phosphate, 100 mM; KCl, 27 mM; NaCl, 1.37 M, pH 7.4 after 1X dilution; 0.1% Tween-20) with primary antibodies (diluted 1:1000) to p-S6RP^S240/244^ (CST 2215), S6RP (CST 2217) and glyceraldehyde-3-phosphate dehydrogenase (GAPDH, SC-51907). Horseradish peroxidase-conjugated secondary antibody (Santa Cruz Biotechnology, Inc., Heidelberg, Germany) was diluted 1:5000. Bound antibodies were visualized by Fusion Fx7 (Fisher Bioblock Scientific, Waltham, MA, USA) using Immobilon^TM^ Western (Millipore Corporation, Billerica, MA, USA), an enhanced chemiluminescence detection kit. The resulting bands were analyzed and quantified by ImageJ® 1.51j software (National Institutes of Health, Bethesda, MD, USA). GAPDH served as a loading control. Each membrane was reused twice after stripping in glycine buffer (6.6 mol/L, pH 2) at 56°C for 30 min. Each experiment was repeated 2 times.

**Sequencing**

Quantity and purity of double-stranded cDNA were verified by GATC Biotech using an Agilent 2100 Bioanalyzer (Agilent Technologies Inc., Waldbronn, Germany). Sequencing libraries were constructed from double-stranded cDNA samples according to the Illumina Genome Analyzer II protocol [2], followed by direct cDNA sequencing in paired end mode with a read length 2 x 125 bp. Then, 30 million read pairs were generated for each sample and FASTQ sequence files were generated by CASAVA. All raw RNAseq sequences were controlled for quality and differential analysis of genes expression were realized.

**NGS RNAseq sequence alignment and quality control pipeline**

Raw RNAseq sequences were controlled for quality using a set of published tools to produce curated reads. Firstly, reads with low quality bases at 5’ and 3’ were trimmed using the Sickle package (Phred cut off 20, max trim size 30 nc) [3]. The SeqPrep package was used to remove sequencing adaptors from raw reads [4]. This package also detected an important proportion of RNA fragments whose R1 and R2 paired-end reads were overlapping and merged them into single-end reads. To keep exploiting those fragment, a home-made python script was developed that split those merged reads into new non-overlapping R1 and R2 paired-end reads. Curated reads were aligned using TOPHAT2 (aka BOWTIE2) on both the UCSC hg19 reference genome and transcriptome [5]. Finally, we applied a post-alignment quality control of aligned reads by removing reads with mapping scores lower than 20 using Samtools [6]. PCR duplicate reads were identified and removed using Picard MarkDuplicates (<https://broadinstitute.github.io>). Read counts were performed using the Python module HTSeq [7]. For clinical samples, only one sample per condition was sequenced which was not statistically congruent with differential expression analysis. We avoided this problem by splitting FASTQ files into three equivalent parts to obtain three subsamples per condition.

**Differential analysis**

Transcript count data were normalized according the VOOM method, which transforms raw count values to log2-counts per million (logCPM), estimates the mean-variance relationship and uses this relationship to compute appropriate observational-level weights [8]. The RNAseq differential gene expression between groups of samples was performed using the statistical t-test from the R package LIMMA, which calculates fold changes and nominal p-values related to each gene starting from raw expression values and the normalization weights produced by VOOM [9]. The set of nominal p-values from each test were adjusted according to the Benjamini-Hochberg adjustment [10]. We defined the significantly up- or down-regulated transcripts using an FDR threshold of 0.05. The fold-change used to further filter the differential gene expression was set to a minimum value of 2. The differential expression status of all genes presenting significant variations is summarized in Supplementary Table S1, S2, S4 and S5.

**Gene Set-Enrichment Analysis**

MSigDB [11] and Ingenuity Pathway Analysis (IPA®, QIAGEN Redwood City) were used to identify pathways or gene ontologies in which the genes of an identified group were enriched. With MSigDB, we used the C2 (canonical pathways) and C5 (biological processes) databases. Oncogene and Tumor Suppressor Gene status (approximately 800 known genes) was assigned according to the annotation in Cancer Gene Panel [12, 13]. The functional annotation database DAVID was used to annotate gene lists of interest [14].

**Aldefluor assay**

5 x 10^5^ cells were resuspended in the Aldefluor assay buffer containing the ALDH1 substrate. As a negative control, an aliquot of Aldefluor-exposed cells was immediately quenched with diethylaminobenzaldehyde (DEAB) which is a specific ALDH1 inhibitor. Following an incubation of 30 min at 37°C, the cells were washed and sorted as ALDH1^high^ or ALDH1^low^ cells using a FACS Calibur flow cytometer (BD Biosciences). The SKBR3 cell line was used as positive control.

**Sphere-forming assay**

For the sphere formation assay, a total of 1 000 FACS-sorted cells were suspended in a stem-cell-permissive medium containing Dulbecco’s Modified Eagle Medium (DMEM-F12) with N2 supplement (Invitrogen), 20 ng/mL EGF and 20 ng/mL basic fibroblast growth factor (bFGF), plated in nonadherent 24-well plates and incubated at 37°C in a 5% CO2 humidified incubator. After 15 days, spheres were counted and photographed under a light microscope using a x20 objective. The self-renewal ability of cells dissociated from tumorspheres by trypsin/EDTA and seeded in new nonadherent 24-well plates was evaluated after 15 days. For drug treatment experiments, 5-day tumor-spheres grown in nonadherent 24-well plates were treated with 0.3 µM of BEZ235 or 0.01 µM of EPZ011989. After 10 days, the number of tumorspheres was recorded.

**Animal studies**

Once palpable, tumor volumes were calculated using the following formula: length x width2 / 2. After tumors reached approximately 100 mm^3^ in average size, animals were treated by oral gavage (100 µL). BEZ235 and EPZ011989 were prepared by dissolving them in 1 volume of NMP (1-methyl-2-pyrrolidone) and 9 volumes of PEG300 (Sigma-Aldrich, St Quentin Fallavier, France). To study the effect of PI3K/mTOR pathway inhibition on LMS tumor growth, mice were randomly assigned to receive 40 mg/kg of BEZ235 or vehicle (NMP/PEG300). To study the anti-tumor effect of BEZ235 and EPZ011989 alone or in combination, mice were randomly pretreated with vehicle or 125 mg/kg of EPZ011989 BID for 2 weeks. Then, in the EPZ011989-pretreated group, mice were randomly treated for 3 weeks with 40 mg/kg of BEZ235 or 125 mg/kg of EPZ011989 BID. Tumor progression was analyzed with GraphPad Prism software, and Kaplan-Meier curve analysis was used to compare individual tumor doubling rates. Formalin-fixed paraffin-embedded tumor sections underwent immunohistochemical analysis for expression of different proteins using standard protocols.

For xenograft experiments in extreme limiting dilution assay (ELDA), 3,000 to 300 FACS-sorted cells were subcutaneously injected with Matrigel into the right (for ALDH1^high^ cells) and the left (for ALDH1^low^ cells) flank of NSG mice (NOD-scid IL2RG^-/-^); tumor size was recorded once a week.

**Immunohistochemistry**

Immunohistochemical methods were described previously [15]. Sections of tumor samples and LMS cell line pellets were incubated with either anti-Ki-67 (Ventana 790-4286; 1:100), anti-p-S6RP^ser240/244^ (CST 5364; 1:100), anti-H3K27Me3 (CST 9733S; 1:100), anti-ALDH1 (Sigma-Aldrich SAB1403542; 1:100) or anti-SOX2 (Ventana 760-4621; 1:100). To confirm a negative labeling in tissues, we used endothelial cells as a positive control. Tissue imaging was conducted with an Olympus CKX41 (x100) using image capture cellSens Entry software version 1.14 for Windows (Olympus, Rungis, France). A pathologist estimated the immunoreactivity signal corresponding to the target expression level based on the percentage of positively stained cells. IHC score was determined by adding the results of multiplication of the percentage of cells with staining intensity ordinal value (scored from 0 for “no signal” to 3 for “strong signal”).

**Statistics**

For analysis of overall survival according to expression levels of ALDH1, the cut-off date for statistical analysis of baseline demographic data and clinical outcome was the 03/31/2018. Survival rates were estimated using the Kaplan–Meier method. Descriptive statistics were used to show the distribution of variables in the population. Differences between groups were evaluated by Chi square test or Fisher’s exact test for categorical variables and Student’s test for continuous variables. Prognostic factors were planned to be identified by univariate and multivariate analyses using a Cox regression model. Variables tested in univariate analysis included: age, gender, primary tumor location, tumor depth, tumor size, grade, and ALDH1 IHC score. Variables associated with a p-value < 0.05 in the univariate analysis were planned to be included in the multivariate analysis. Analyses were performed using SPSS 18.0 statistical software (IPSS Inc., Chicago, USA). All statistical tests were two-sided, and p < 0.05 indicated statistical significance.

For experiments, quantification values represent the means of three or more experiments ± SEM. Differences in mean values between two groups were analyzed by two-tailed Student’s t-tests. Differences in three or more than three groups were analyzed by one-way or two-way ANOVA, followed by the post hoc Tukey’s honest significant difference test. Log-rank (Mantel-Cox) tests were used to compare Kaplan-Meier curves using GraphPad Prism. Furthermore, ELDA analyses were performed using the “limdil” function of the R-package “StatMod” [16, 17]. Cancer-initiating cell frequencies with 95% confidence intervals were estimated and are expressed in 1/stem cell frequency. CSC frequencies were compared between groups (e.g. presence or absence of ALDH1 activity) using likelihood ratio tests. A *p < 0.05* was required for results to be considered statistically significant.

# **Supplementary Figures**

**
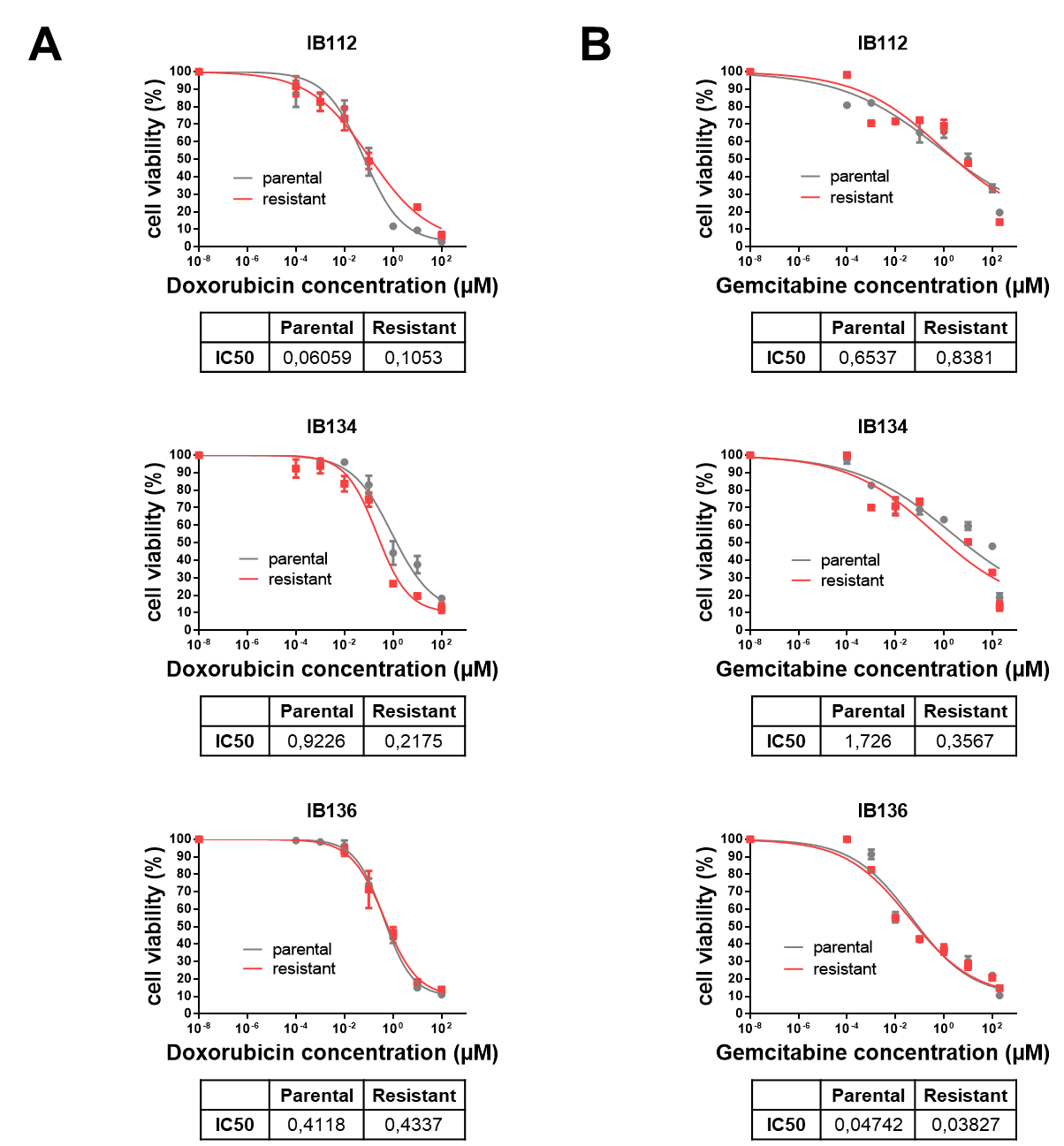
**

**Figure S1.** Effect of doxorubicin and gemcitabine on cell growth in parental and resistant leiomyosarcoma (LMS) cell lines. **A,** Growth curves indicating growth inhibition of the three parental (IB112, IB134 and IB136) and resistant (resIB112, resIB134 and resIB136) LMS cell lines after doxorubicin treatment for 72 hours. **B,** Growth curves indicating growth inhibition of LMS cell lines after gemcitabine treatment for 72 hours. IC_50_ is indicated in µM. Data are presented as the mean ± SEM of three independent experiments.

**
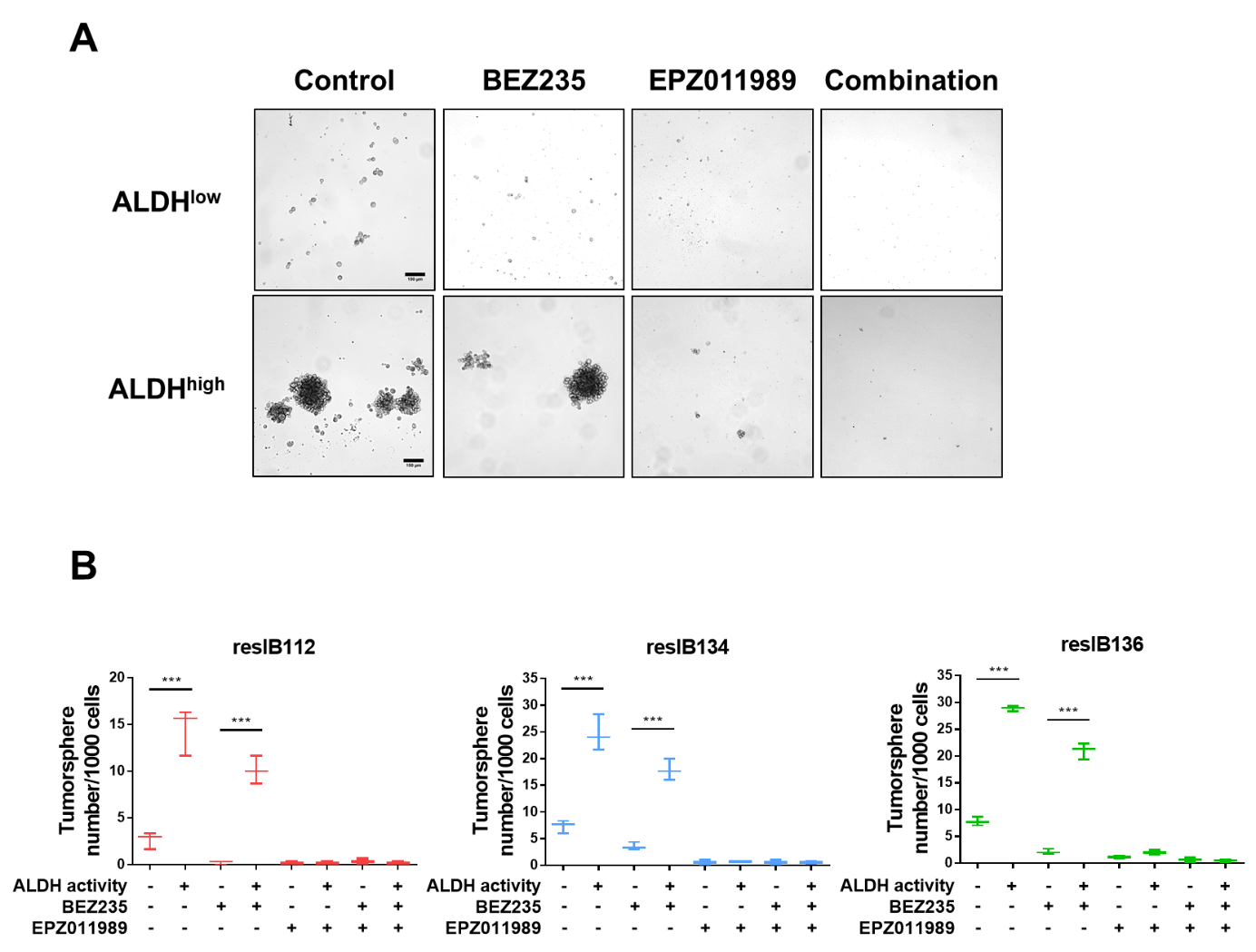
**

**Figure S2.** Effect of BEZ235 and EPZ011989 treatment on tumorsphere formation. **A,** Resistant LMS cells were sorted by FACS based on ALDH1 activity and the positive and negative subpopulations of sorted cells were submitted to *in vitro* tumorsphere assays. Representative images of phase contrast microscopy of tumorspheres formed by resIB136 cells treated with BEZ235 treatment (0.3 µM) or EPZ011989 treatment (0.01 µM), alone or in combination, after 15 days of culture in nonadherent conditions *in vitro*. Scale bars, 150µm. **B,** Plots (min and max) represent the number of tumorspheres formed after 15 days of BEZ235 and EPZ011989 treatment, alone or in combination, in ALDH1^high^ and ALDH1^low^ subpopulation. ****p* < 0.001, one-way ANOVA.

# **Supplementary tables**

**Table S1.** Clinical parameters of the two independent cohorts of patients with LMS.

**
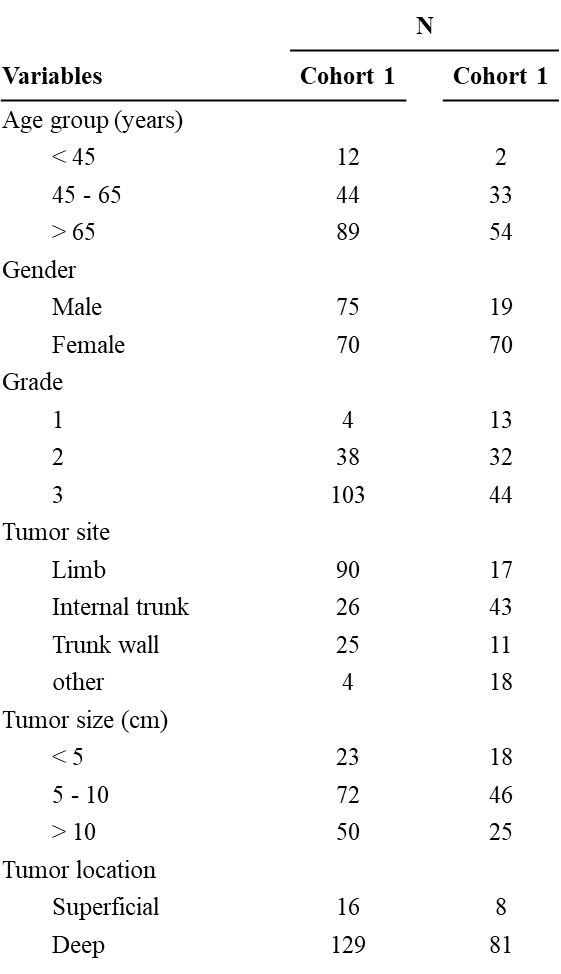
**

**Table S2.** List of up-regulated genes in resIB136 xenografts (with fold-change and *p-values*).

**
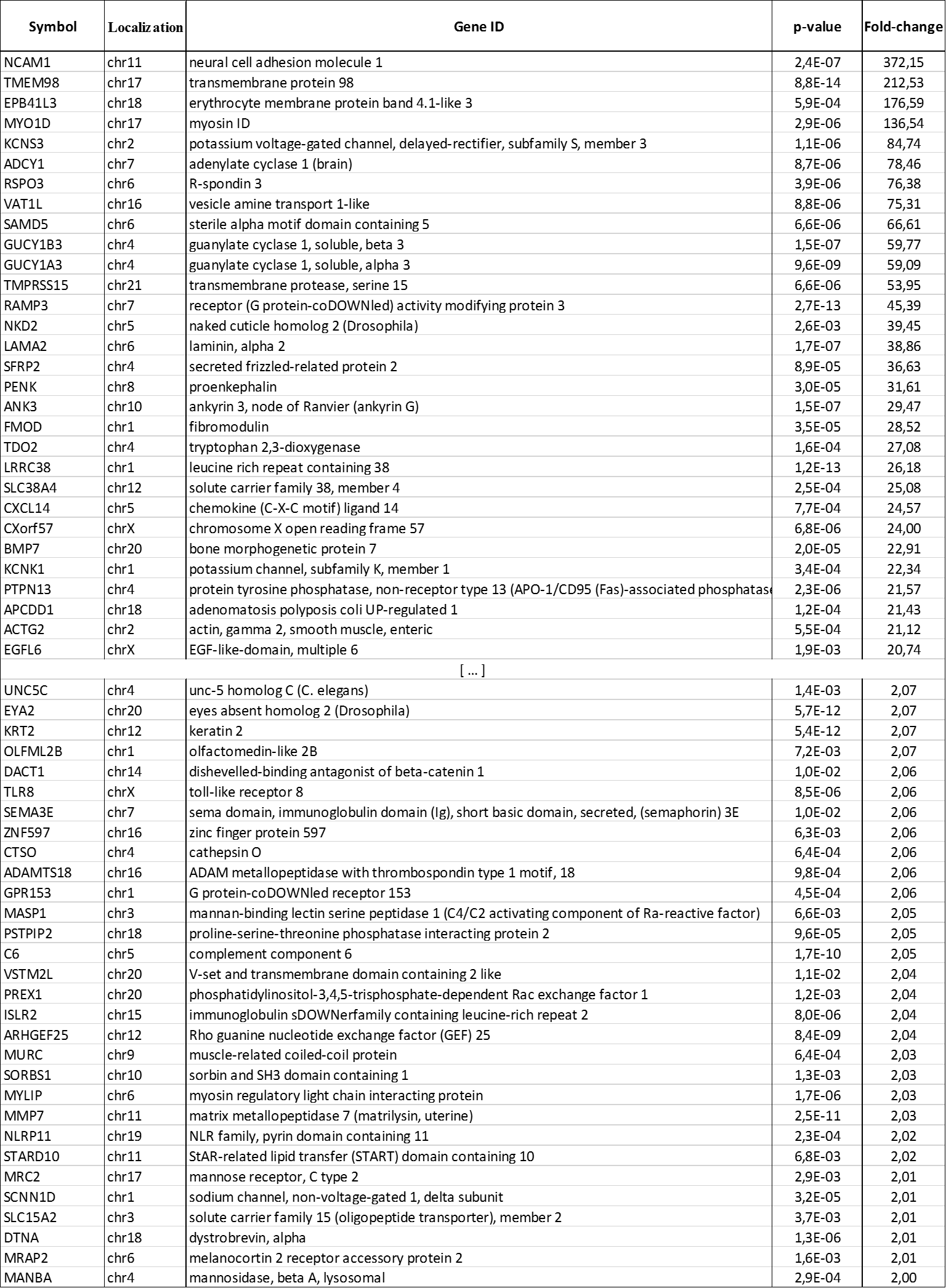
**

**
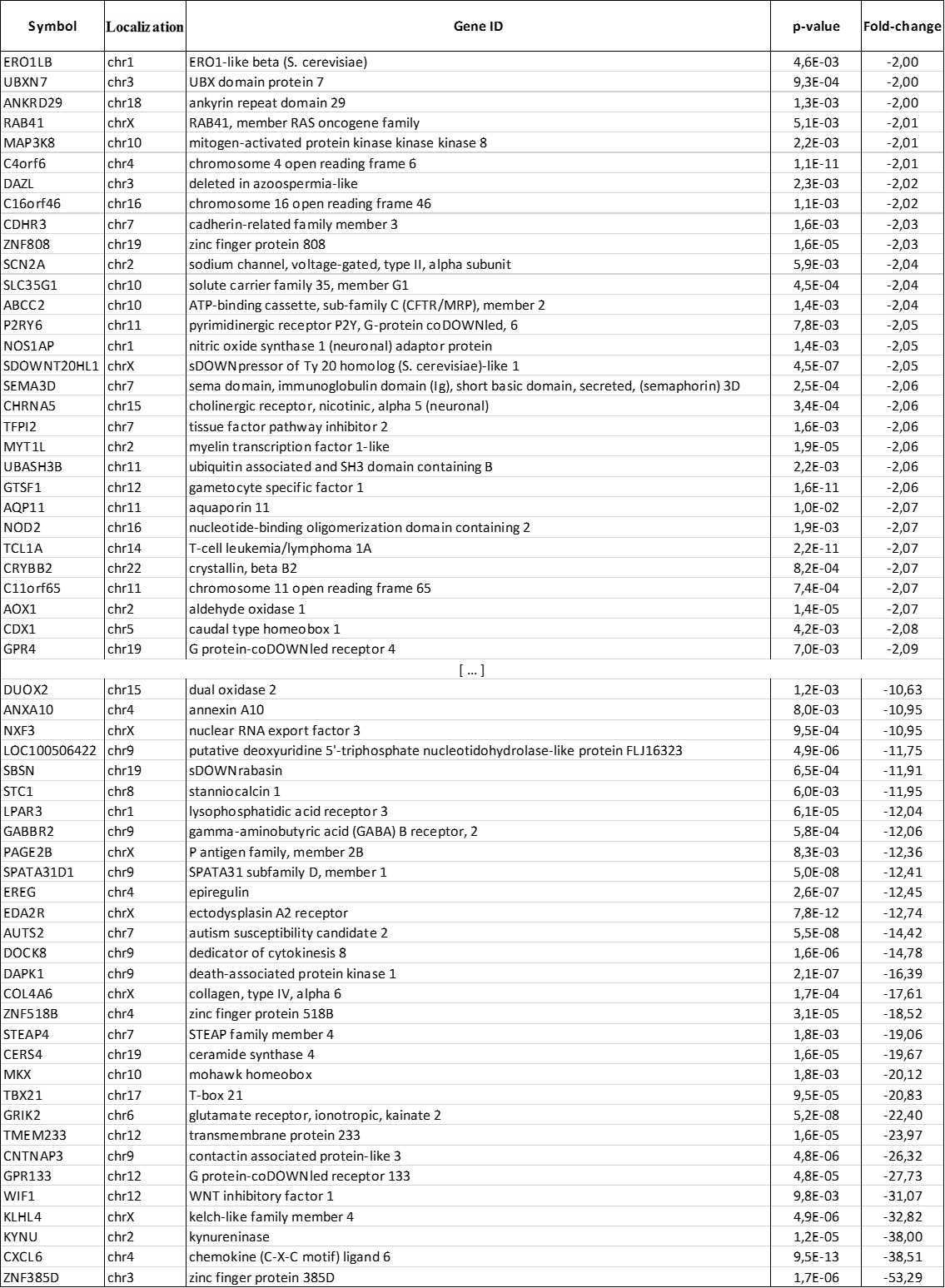
Table S3.** List of down-regulated genes in resIB136 xenografts (with fold-change and *p-values*).

**Table S4.** List of enriched pathways in resIB136 xenografts (with *p-values*).

**Table S5.** Enrichment of cancer stem cell-like genes in resIB136 xenografts (with fold-changes and *p-values*).


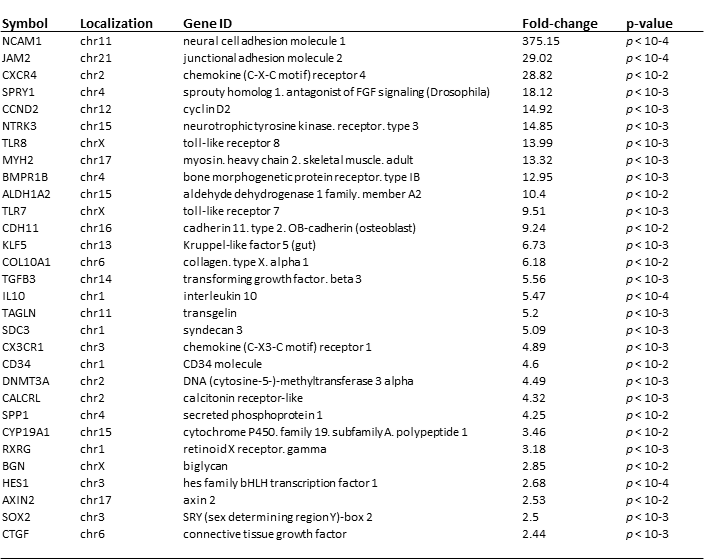


**Table S6.** Cancer-initiating cell frequencies determined on FACS-sorted cells according to ALDH1 activity after tumor xenografts in limiting dilution in NSG mice.


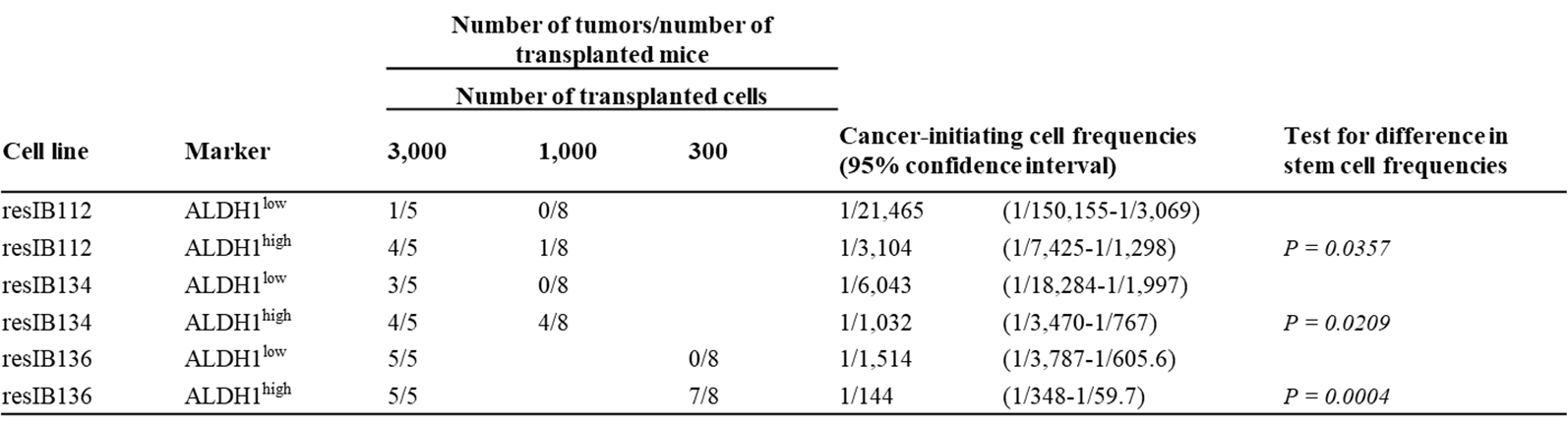


**
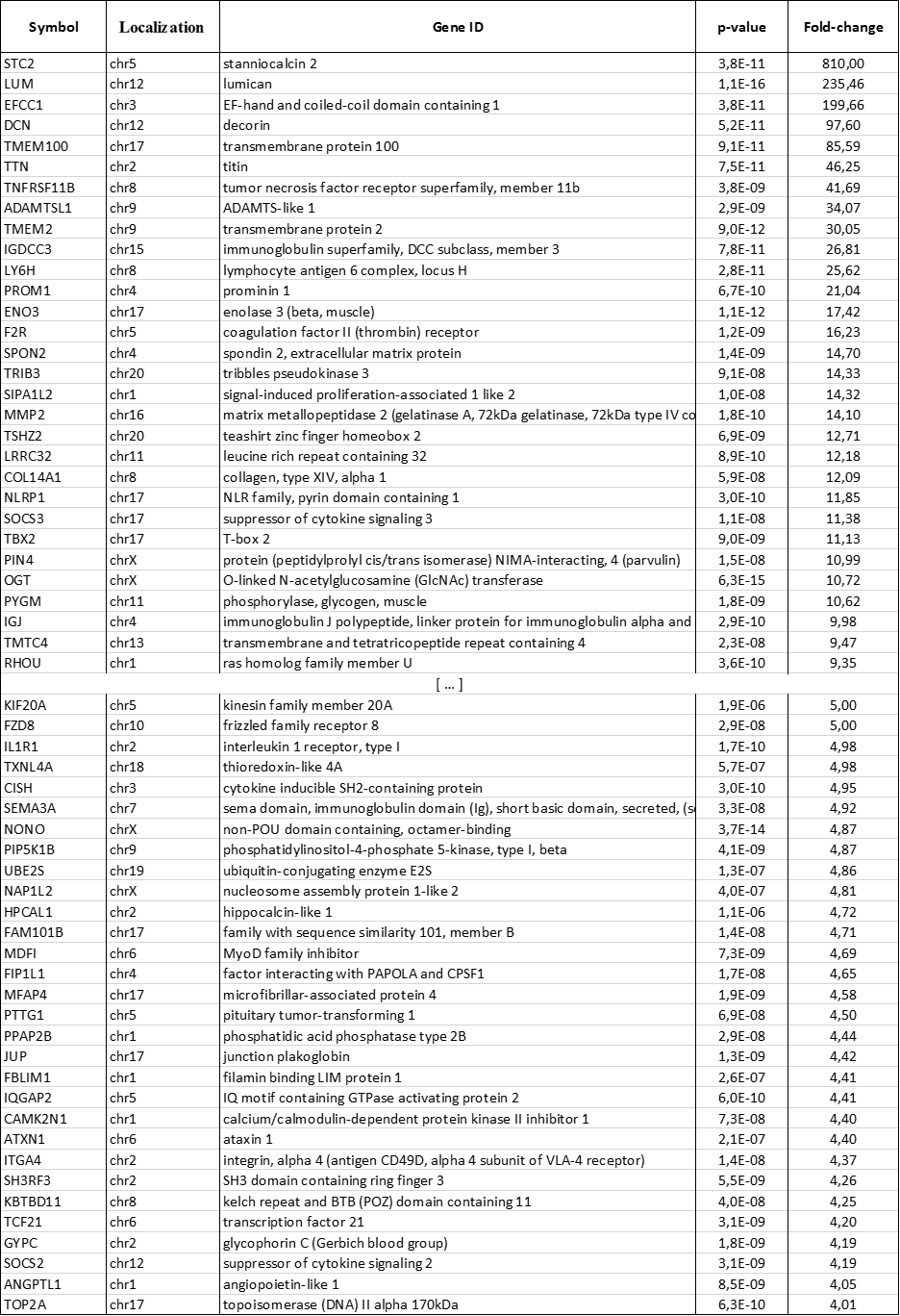
Table S7.** List of up-regulated genes in clinical sample (with fold-change and *p-values*).

**Table S8.** List of down-regulated genes in clinical sample (with fold-change and *p-values*).

**
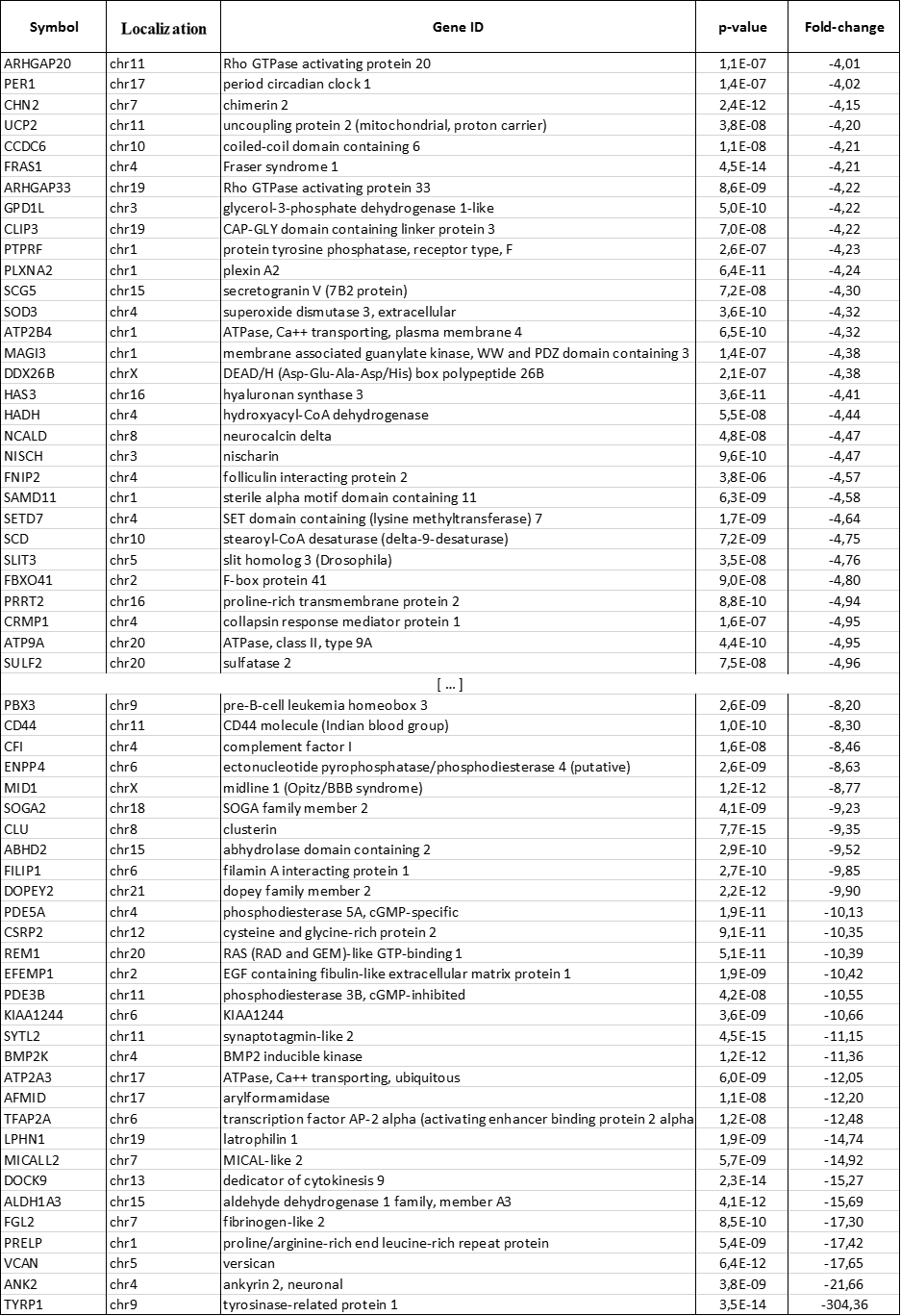
**

**Table S9.** List of enriched pathways in clinical sample (with *p-values*).

**Table S10.** Enrichment of cancer stem cell-like genes in clinical sample (with fold-change and *p-values*).


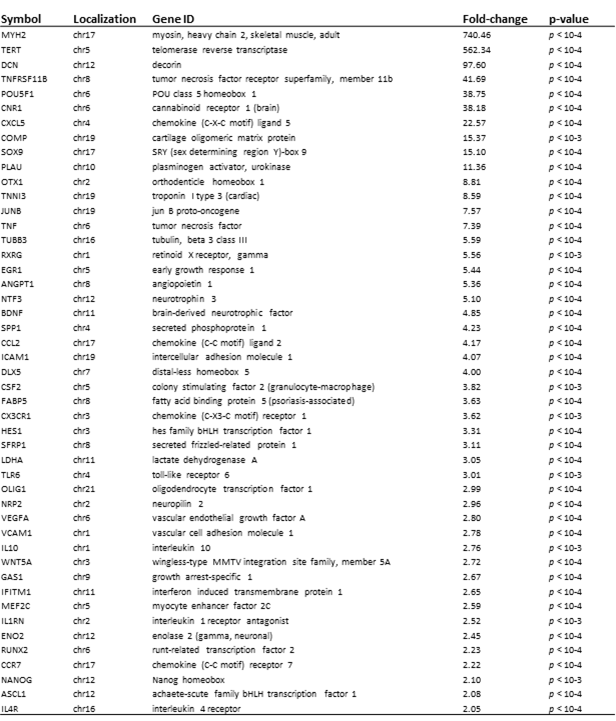


**References**

1. Harlow E, Lane D. Lysing tissue-culture cells for immunoprecipitation. CSH Protoc. 2006. doi:10.1101/pdb.prot4531.

2. Wilhelm BT, Landry J-R. RNA-Seq-quantitative measurement of expression through massively parallel RNA-sequencing. Methods San Diego Calif 2009; 48(3):249–257.

3. najoshi. sickle: Windowed Adaptive Trimming for fastq files using quality, 2017.

4. John JS. SeqPrep: Tool for stripping adaptors and/or merging paired reads with overlap into single reads, 2017.

5. Langmead B, Salzberg SL. Fast gapped-read alignment with Bowtie 2. Nat. Methods 2012; 9(4):357–359.

6. Li H, Handsaker B, Wysoker A et al. The Sequence Alignment/Map format and SAMtools. Bioinforma. Oxf. Engl. 2009; 25(16):2078–2079.

7. Anders S, Pyl PT, Huber W. HTSeq--a Python framework to work with high-throughput sequencing data. Bioinforma. Oxf. Engl. 2015; 31(2):166–169.

8. Law CW, Chen Y, Shi W, Smyth GK. voom: Precision weights unlock linear model analysis tools for RNA-seq read counts. Genome Biol. 2014; 15(2):R29.

9. Ritchie ME, Phipson B, Wu D et al. limma powers differential expression analyses for RNA-sequencing and microarray studies. Nucleic Acids Res. 2015; 43(7):e47.

10. Benjamini Y, Hochberg Y. Controlling the False Discovery Rate: A Practical and Powerful Approach to Multiple Testing. J. R. Stat. Soc. Ser. B Methodol. 1995; 57(1):289–300.

11. Subramanian A, Tamayo P, Mootha VK et al. Gene set enrichment analysis: a knowledge-based approach for interpreting genome-wide expression profiles. Proc. Natl. Acad. Sci. U. S. A. 2005; 102(43):15545–15550.

12. Vogelstein B, Papadopoulos N, Velculescu VE et al. Cancer genome landscapes. Science 2013; 339(6127):1546–1558.

13. Singh RR, Patel KP, Routbort MJ et al. Clinical massively parallel next-generation sequencing analysis of 409 cancer-related genes for mutations and copy number variations in solid tumours. Br. J. Cancer 2014; 111(10):2014–2023.

14. Huang DW, Sherman BT, Lempicki RA. Bioinformatics enrichment tools: paths toward the comprehensive functional analysis of large gene lists. Nucleic Acids Res. 2009; 37(1):1–13.

15. Fourneaux B, Chaire V, Lucchesi C et al. Dual inhibition of the PI3K/AKT/mTOR pathway suppresses the growth of leiomyosarcomas but leads to ERK activation through mTORC2: biological and clinical implications. Oncotarget 2017; 8(5):7878–7890.

16. Bonnefoix T, Bonnefoix P, Callanan M et al. Graphical representation of a generalized linear model-based statistical test estimating the fit of the single-hit Poisson model to limiting dilution assays. J. Immunol. Baltim. Md 1950 2001; 167(10):5725–5730.

17. Hu Y, Smyth GK. ELDA: Extreme limiting dilution analysis for comparing depleted and enriched populations in stem cell and other assays. J. Immunol. Methods 2009; 347(1):70–78.
